# Supplementary material for: Compensating affected parties necessary for rapid coal phase-out but expensive if extended to major emitters
Source: Nat Commun. 2024 May 7;15:3742. doi: 10.1038/s41467-024-47667-w (PMC11076460; doi:10.1038/s41467-024-47667-w)
Supplement: Supplementary file 1 — Supplementary Information [file 41467_2024_47667_MOESM1_ESM.pdf]

Supplementary information
For the article

Compensating affected parties necessary for rapid coal phase-out but expensive if extended to major emitters

Lola Nacke[a], Vadim Vinichenko[a], Aleh Cherp[b,c], Avi Jakhmola[a], Jessica Jewell[a,d,e]

[a] Department of Space, Earth and Environment, Chalmers University, Gothenburg, Sweden. [b] Department of Environmental Science and Policy, Central European University, Vienna, Austria. [c] International Institute for Industrial Environmental Economics, Lund University, Lund, Sweden. [d] Centre for Climate and Energy Transformations and Geography Department, University of Bergen, Bergen, Norway. [e] Advancing Systems Analysis, International Institute for Applied Systems Analysis, Laxenburg, Austria.

Table of Contents

SUPPLEMENTARY FIGURES **2**

SUPPLEMENTARY TABLES **7**

SUPPLEMENTARY NOTES **24**

SUPPLEMENTARY NOTE 1. JUST ENERGY TRANSITION PARTNERSHIPS 24
SUPPLEMENTARY NOTE 2. UNCERTAINTY AND ROBUSTNESS ANALYSIS 25
SUPPLEMENTARY NOTE 3. REGRESSION ANALYSIS 30
SUPPLEMENTARY NOTE 4. JUST TRANSITIONS FOR COAL PHASE-OUT 41

REFERENCES **45**

Supplementary Figures

Supplementary Figure 1. Overview of Methods for building dataset.

Countries with coal phase-out pledges are based on Vinichenko et al[1] (43 countries). Countries which have no coal capacity at the time of making the phase-out pledge and countries which do not have a year associated with their coal phase-out pledge are excluded (Methods, Supplementary Table 3). Out of these 43 countries, 23 also have a compensation policy, identified using a systematic review and expert consultations (Methods). South Africa also receives international compensation through their JETP but does not have a coal phase-out pledge.

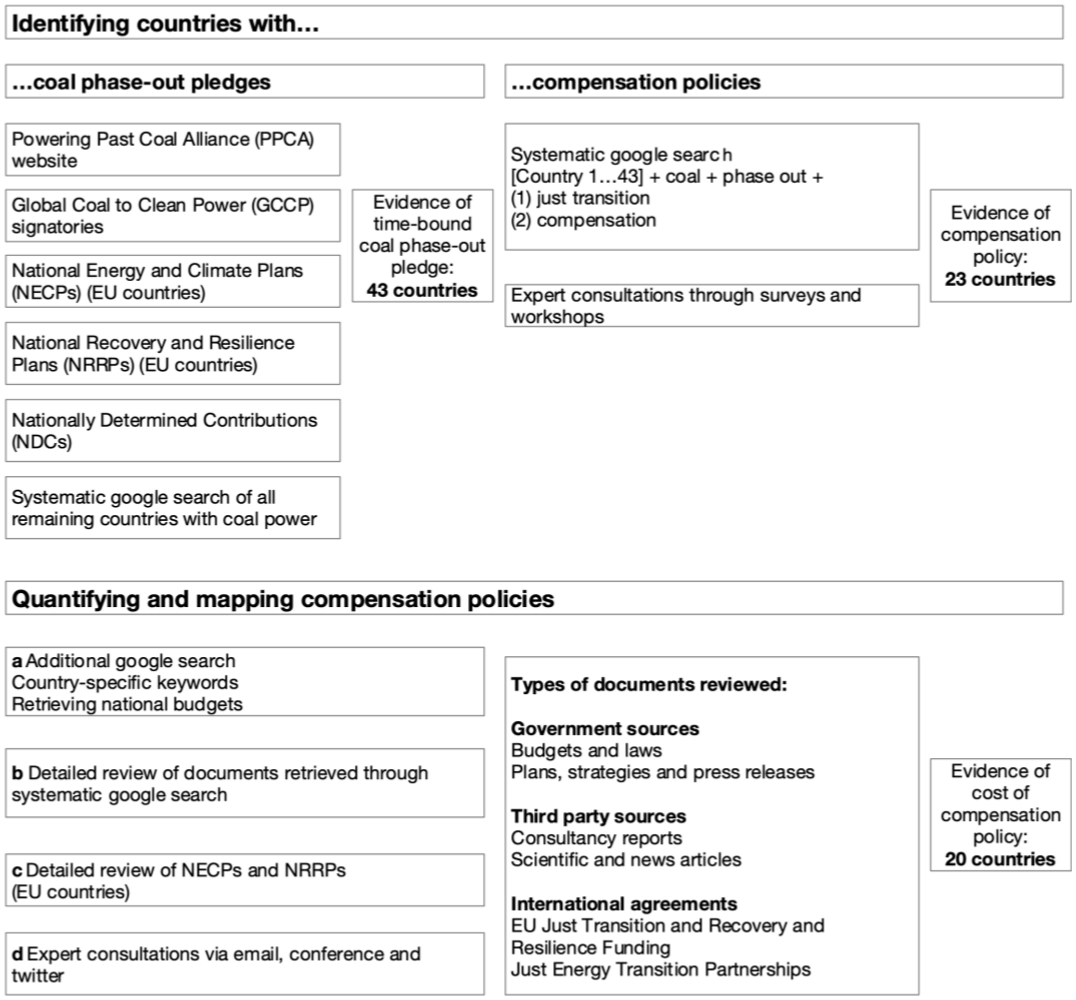

Supplementary Figure 2. Types of compensation from international and domestic funding.

Bars show central compensation estimates, error bars show the lower and upper compensation estimates for each type of support due to uncertainties in compensation policies (Methods, Supplementary Table 1). Lighter shaded areas show international compensation, brighter shaded areas show domestic compensation. All JETP-compensation is international, all support for coal power plant and mine closures, renewables capacity and low-carbon infrastructure, unemployment benefits and retraining as well as regional development to SMEs is domestic. The EU just transition fund (international) is included under “Regional development”, some of this funding may be used by regions to support the local economy, which may ultimately support employment retraining or renewables development.

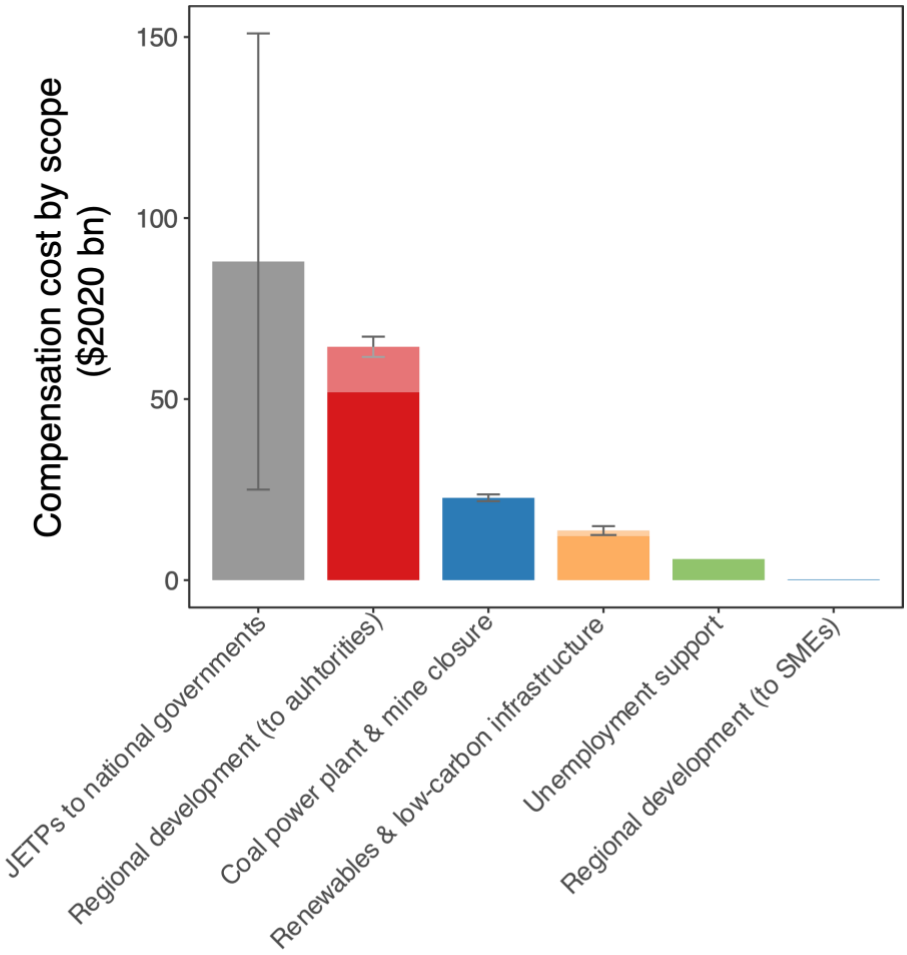

Supplementary Figure 3. Average annual compensation compared to coal production subsidies. **a)** Compensation estimates represent the central estimate (Table 1, Supplementary Table 1, Methods). Annual compensation is the total compensation divided by the number of years between the phase-out pledge and the pledged phase-out date. Coal production subsidy data compiled from the OECD[2] and from IISD[3] respectively. The main estimate excludes investment in state-owned companies since in contrast to compensation payments and other subsidies, governments expect a return to an investment. Subsidy data could not be identified for Vietnam, Bulgaria, Croatia and Romania. **b)** Median estimate of potential coal phase-out compensation cost for India and China to stay on 1.5°C-, 2°C- and 2.5°C-compatible pathways based on the average compensation per ton avoided emissions. Coal production subsidy data compiled from the OECD[2] and from IISD[3] respectively. The main estimate excludes investment in state-owned companies (also in Figure 4) since in contrast to compensation payments and other subsidies, governments expect a return to an investment. In Germany, coal production subsidies are in fact comparable to compensation in contrast to China and India where they are still far smaller than the estimated compensation.

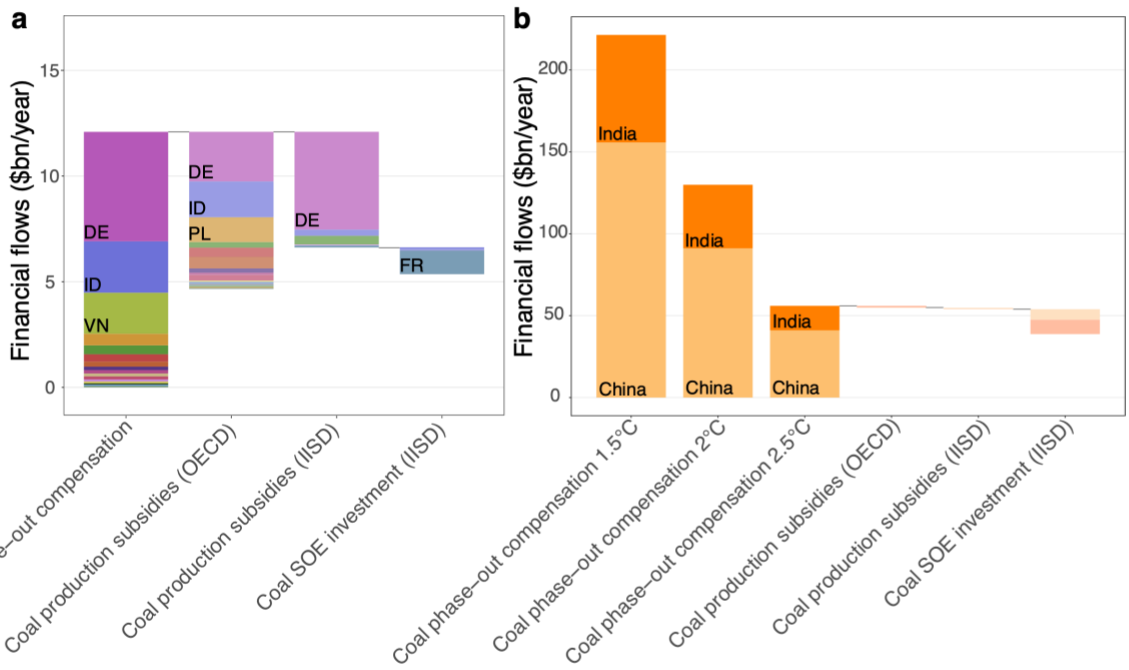

Supplementary Figure 4. Total compensation in international context.

Compensation estimates represent the central estimate (Table 1, Supplementary Table 1, Methods). Cumulative financial flows for Official Development Assistance (ODA), coal production subsidies, and the COP climate pledge are calculated as the average between the IQR of the duration of coal phase-out in China and India in IPCC 1.5°C-, 2°C-, and 2.5°C-compatible pathways. See Supplementary Table 7 for a description of and sources for each financial flow, as well as for uncertainty ranges. South Africa is excluded from this figure because there is no phase-out date, so it is not possible to estimate compensation over the entire phase-out period by extrapolation in the same way as for Indonesia and Vietnam (see Methods, Table 2, Supplementary Table 1).

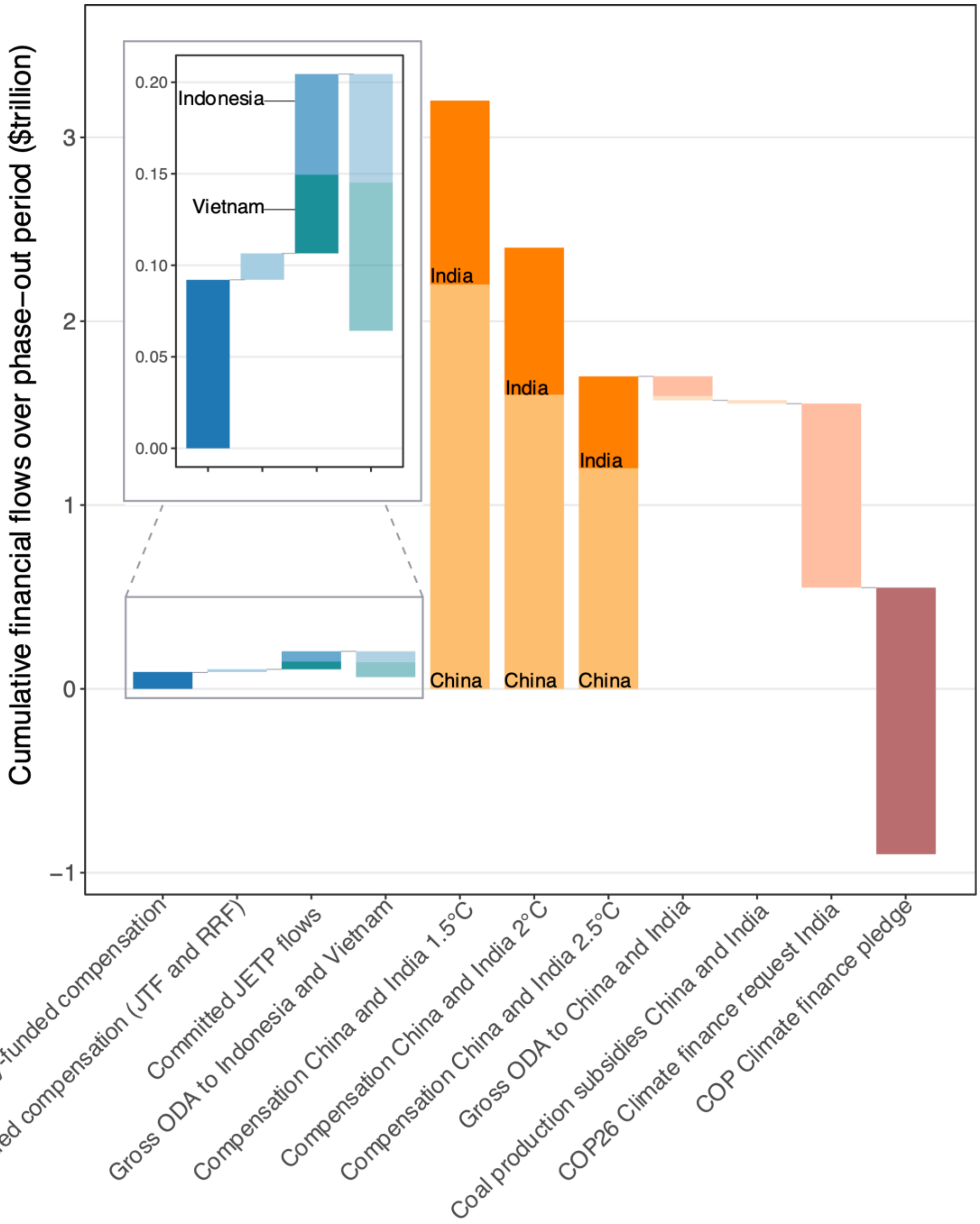

Supplementary Figure 5. Annual coal phase-out compensation in domestic and international context. **(a)** Blue bars represent the central estimate for coal phase-out compensation policies (dark blue from domestic and light blue from international sources) and error bars the lower and upper compensation estimates due to uncertainties in compensation policies and coal phase-out pledges. The uncertainty ranges for EU and OECD countries are based on uncertainties from empirical compensation cost (for example, where compensation could not be confirmed in official government documentation - Methods). For JETP-recipients, Indonesia and Vietnam, uncertainty ranges represent annual rates of currently pledged compensation (which is pledged over 3-5 years, see Methods, Table 2). Annual compensation is the total compensation divided by the number of years between the phase-out pledge and the pledged phase-out date normalized to GDP in the year each phase-out pledge was made. Orange bars represent the median estimate of potential coal phase-out compensation cost for India and China to stay on 1.5°C-, 2°C- and 2.5°C-compatible pathways based on the average compensation per ton avoided emissions. The error bars represent the uncertainty range, which is calculated based on the ten best-performing regression models, the confidence interval in each model, and range of avoided emissions across IPCC pathways (Methods, Supplementary Table 1). Compensation is normalized to GDP in 2021[4] (Methods). **(b)** Central estimates for planned domestic (dark blue bars) and international (light blue and green bars) compared to Official Development Assistance[5] for JETP countries, scenario-based compensation (orange bars), coal production subsidies in China and India[2], the climate finance request by India's Prime Minister at COP26[6], and the climate finance pledge first made at COP15[7] (Methods, Supplementary Table 7, Supplementary Figure 4).

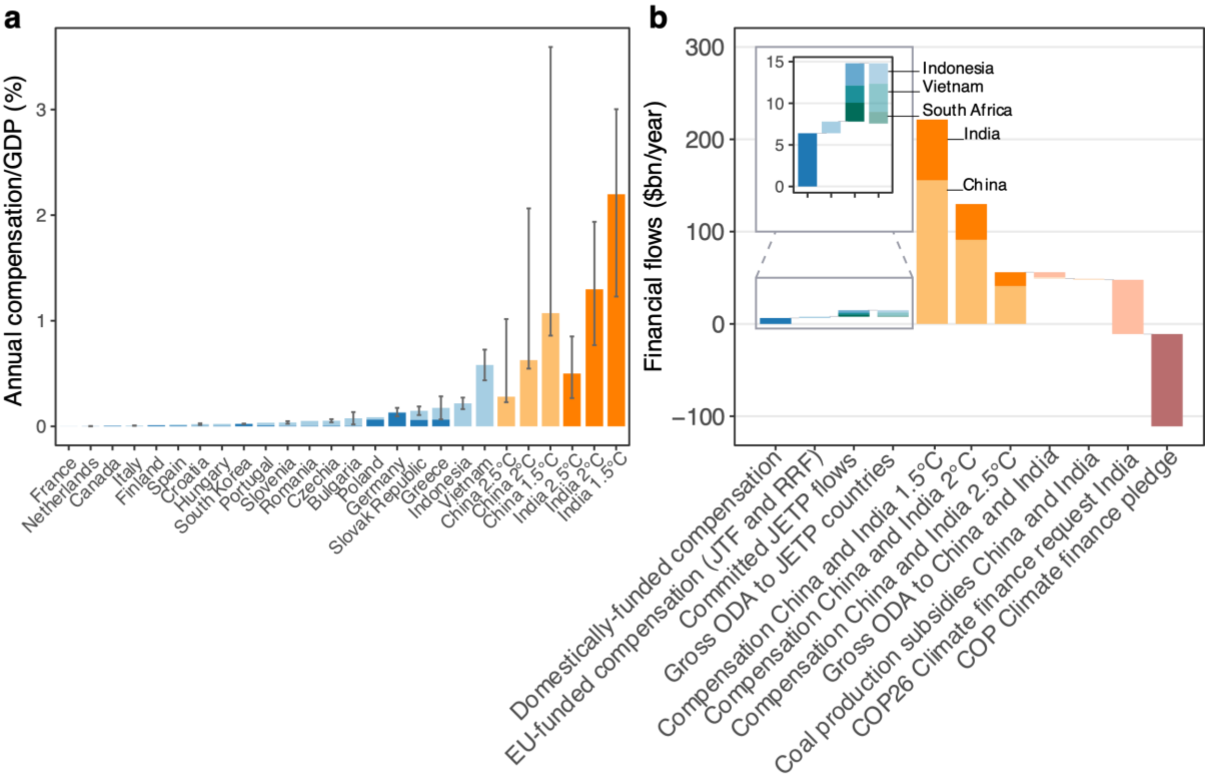

Supplementary Tables

Supplementary Table 1. Detailed compensation estimates and sources.

EU Just Transition funding is dependent on approval of territorial just transition plans – if not stated otherwise in the table, these plans have been approved.

	Upper/ lower estimate	Total estimate ($\$_{2020}$ mln)	Estimate for each mechanism ($\$_{2020}$ mln)	Description of support (from original documents)	Type of support (coded)
**Bulgaria**	Lower estimate	260	260	Transfer to "design, build and commission infrastructure adequate for transmission of hydrogen and low-carbon gaseous fuels". Pledge confirmed in Recovery and Resilience Facility.[8]	Renewables and low-carbon infrastructure
	Upper estimate	1,480	1,480	EU transfer to coal regions to support structural development. Dependent on approval of Territorial Just transition Plan[9–11].	Regional development
**Canada**	Estimate	1,200	30	Transfer to coal regions, focusing on skills development and economic diversification. Pledge confirmed in national budget.[12,13]	Unemployment support
			110	Transfer to coal regions, to invest in infrastructure. Pledge confirmed in national budget.[12,13]	Regional development
			2	Transfer to coal regions to invest in clean energy and transmission/integration projects and to help territories to identify options to reduce reliance on coal-fired electricity. Allocation may also benefit non-coal regions.[14]	Renewables and low-carbon infrastructure
			870	Transfer to TransAlta, ATCO, and Capital Power, the major coal utilities in Alberta, for foregone profits. Regional phase-out in Alberta ahead of national phase-out[15].	Coal power plant closure
			4	Transfer to municipalities, for economic diversification plans and feasibility studies. Regional phase-out in Alberta ahead of national phase-out.[15]	Regional development
			30	Transfer to laid-off workers. Regional phase-out in Alberta ahead of national phase-out.[15]	Unemployment support
			150	Transfer to help coal and indigenous communities adjust to coal phase-out. Regional phase-out in Alberta ahead of national phase-out.[16]	Regional development
**Croatia**	Lower estimate	110	90	EU transfer to support regions negatively affected by climate transition measures[17]. The original eligibility analysis describes that one eligible region hosts a coal power plant[10]. Since the exact flows allocated to each region and purpose are not specified, we assume that half of the overall EU funding is directed at the coal phase-out as the lower estimate[18].	Regional development
			20	Share of national co-financing to the EU JTF[18].	Regional development
	Upper estimate	110	100	Since the exact flows allocated to each region and purpose are not specified, we assume that the other half is also directed at the coal phase-out as the upper estimate[18].	Regional development

	Upper/ lower estimate	Total estimate ($_{2020}$ mln)	Estimate for each mechanism ($_{2020}$ mln)	Description of support (from original documents)	Type of support (coded)
			10	Share of remaining national co-financing to the EU JTF[18].	Regional development
Czechia	Lower estimate	1,230	210	Transfer to increase installed capacity of sources of photovoltaic energy. Pledge confirmed in Recovery and Resilience Facility.[19]	Renewables and low-carbon infrastructure
			70	Transfer to modernize heat distribution. Pledge confirmed in Recovery and Resilience Facility.[19]	Renewables and low-carbon infrastructure
			810	EU transfer to support regions negatively affected by climate transition measures[10,20,21]. This includes three regions that host coal but also other carbon-intensive industries[20]. Since the exact flows allocated to each region and purpose are not specified, we assume that half of the overall EU funding is directed at the coal phase-out as the lower estimate.[18]	Regional development
			140	Share of national co-financing to the EU JTF[18].	Regional development
	Upper estimate	1,890	1,600	Since the exact flows allocated to each region and purpose are not specified, we assume that all EU funding is directed at the coal phase-out as the upper estimate[18,20,21].	Regional development
			290	Share of national co-financing to the EU JTF[18].	Regional development
Finland	Estimate	280	180	Transfer to companies for investments in alternatives to coal. Pledge from national budget.[22]	Coal power plant closure
			100	Transfer to companies and municipalities to promote renewables and improve energy efficiency. Pledge from government website.[23]	Renewables and low-carbon infrastructure
France	Estimate	80	30	Transfer to laid-off workers to compensate for job loss. Pledge confirmed in national budget[24].	Unemployment support
			50	Transfer to regions to support with the costs of coal plant closure. Pledge confirmed in coal phase-out plan.[24,25]	Regional development
Germany	Lower estimate	65,540	640	Transfer to hard coal companies to compensate for closure of power plants (through auctions). Pledge confirmed in coal phase-out law[26], lower estimate based on empirical data for first five auctions from[27].	Coal power plant closure
			2,000	Transfer to Lausitz Energie Kraftwerk AG for costs of mine closures (negotiated amount of compensation). Pledge confirmed in coal phase-out law.[26]	Coal mine closure
			2,970	Transfer to RWE AG for costs of mine closures (negotiated amount of compensation). Pledge confirmed in coal phase-out law. [26]	Coal mine closure
			3,430	Transfer to Combined Heat and Power (CHP) plant operators, to support coal alternatives. Pledge confirmed in coal phase-out law. [26]	Coal power plant closure
			5,710	Transfer to laid-off workers to compensate for job loss. Pledge confirmed in coal phase-out law.[26]	Unemployment support
			15,990	Transfer to brown coal regions for structural development. Pledge confirmed in structural development law.[28]	Regional development

	Upper/ lower estimate	Total estimate ($\$_{2020}$ mln)	Estimate for each mechanism ($\$_{2020}$ mln)	Description of support (from original documents)	Type of support (coded)
			29,700	Transfer to hard and brown coal regions for structural development Pledge confirmed in structural development law.[28]	Regional development
			1,250	Transfer to hard coal and former brown coal mining regions for structural development. Pledge confirmed in structural development law[28].	Regional development
			2,370	EU transfer to support affected regions of the climate transition[10,29]. This includes four regions affected by the coal phase-out[29]. We exclude transfers to support the phase-out of an oil refinery[18].	Regional development
			1,480	Share of national co-financing to the EU JTF[18].	Regional development
	Upper estimate	2,280	2,280	Transfer to hard coal companies to compensation for closure of power plants (through auctions). Pledge confirmed in coal phase-out law[26], upper estimate based on maximum amount reported in non-governmental source[30].	Coal power plant closure
	Excluded			Potential future transfer to energy intensive industries to compensate for rising electricity costs due to the coal phase-out[26]. We were not able to retrieve the announced legislation (Förderrichtlinie) or estimate a specific amount of such compensation.	
Greece	Lower estimate	1,200	340	National transfer to coal regions for investments in infrastructure. Pledge confirmed in national Just Transition Plan[31].	Regional development
			730	EU transfer to support regions negatively affected by climate transition measures[10]. This includes two regions that host coal industries, as well as oil-powered plants[32]. Since the exact flows allocated to each region and purpose are not specified, we assume that half of the overall EU funding is directed at the coal phase-out as the lower estimate.[18]	Regional development
			130	Share of national co-financing to the EU JTF[18].	Regional development
	Upper estimate	1,960	230	National transfer to coal regions for structural development. From non-governmental report, not confirmed in national budget, law or plan.[33]	Regional development
			730	Since the exact flows allocated to each region and purpose are not specified, we assume that the other half is also directed at the coal phase-out as the upper estimate.[18,32]	Regional development
			140	Share of remaining national co-financing to the EU JTF[18].	Regional development
Hungary	Estimate	320	270	EU transfer to support regions negatively affected by climate transition measures[10,34]. Since the original eligibility analysis and the final allocation news release only mention coal, we assume all EU JTF funding is paid in relation to the coal phase-out[10,18,34].	Regional development
			50	Share of national co-financing to the EU JTF[18].	Regional development

	Upper/ lower estimate	Total estimate ($_{2020}$ mln)	Estimate for each mechanism ($_{2020}$ mln)	Description of support (from original documents)	Type of support (coded)
Indonesia	Lower estimate	31,000	31,000	Own extrapolation of original pledge over the minimum duration of phase-out in line with the GCCP (2023-2040). International transfer to Indonesia to support coal phase-out and decarbonization in line with just transition. Different purposes are outlined, but it is not specified what share of funding will be allocated to each purpose:<br>“Restricting the development of captive coal fired power plants […],Freezing the existing pipeline of planned on-grid coal-fired power plants […],Accelerating the deployment of renewable energy […],Accelerating the widespread deployment of energy efficiency and electrification tools, technologies, and reforms […],Accelerating the development of a vibrant and competitive local industry in renewable energy and energy efficiency, […],identify and support the segments of Indonesia's population most vulnerable to potential negative impacts of the transition, workers and all societal groups with a special focus on women, youth, and vulnerable populations that earn a living in the coal industry or in jobs connected with the coal industry”.<br>Pledge confirmed in international agreement.[35]	JETP to national government<br>*Support coal power plant closure, Renewables and low-carbon development, unemployment support, regional development*
	Upper estimate	78,900	78,900	Own extrapolation of original pledge over the maximum duration of phase-out in line with the GCCP (2023-2049). (Methods, Supplementary Note 1).	JETP to national government<br>*Support coal power plant closure, Renewables and low-carbon development, unemployment support, regional development*
Italy	Lower estimate	870	740	EU transfer to support regions negatively affected by climate transition measures[10,36]. Since the news report documenting the final allocation describes that funding supports coal phase-out in the region of Sulcis Iglesiente, we include this funding in the lower estimate[10,18,36].	Regional development
			130	Share of national co-financing to the EU JTF[18].	Regional development
	Upper estimate	460	390	Since the original eligibility analysis mentions that the region of Taranto also hosts coal industry, we include this funding in the upper estimate[10,18,36].	Regional development
			70	Share of national co-financing to the EU JTF[18].	Regional development
Netherlands	Lower estimate	60	60	Transfer to Vattenfall to compensate for the early closure of the Hemweg plant. Confirmed in EU State Aid case. [37]	Coal power plant closure
	Upper estimate	240	240	Transfer to Riverstone to compensate for early closure of the Onyx power plant. Not confirmed in national documents.[38]	Coal power plant closure
	Excluded	3,350	2,230	Transfer requested by RWE AG to compensate for early closure of power plants, but claim has been denied by The Hague District Court.[39]	

	Upper/ lower estimate	Total estimate ($_{2020}$ mln)	Estimate for each mechanism ($_{2020}$ mln)	Description of support (from original documents)	Type of support (coded)
			1,120	Transfer requested by Uniper to compensate for early closure of power plants, but claim has been denied by The Hague District Court.[39]	
**Poland**	Estimate	15,330	8,200	Transfer to energy company EDM to cover costs of running and decommissioning coal plants. Confirmed in national coal phase-out plan.[40]	Coal power plant closure
			1,280	Transfer to company PGG for support of coal mine closure. Confirmed in national coal phase-out plan.[40]	Coal mine closure
			2,050	Transfer to coal mining company SRK to compensate for costs of coal mine closure. Confirmed in EU State Aid case.[41]	Coal mine closure
			-920	Estimated gain from operation of coal power plants until closure. Confirmed in coal phase-out plan.[40]	
			3,800	EU transfer to support regions negatively affected by climate transition measures[10,42]. Since the original eligibility analysis and the final allocation news release only mention coal, we assume all EU JTF funding is paid in relation to the coal phase-out.[10,18,42]	Regional development
			920	Share of national co-financing to the EU JTF[18].	Regional development
**Portugal**	Estimate	260	180	EU transfer to support regions negatively affected by climate transition measures[10,43]. This includes two regions with coal-fired power plants, and one city with an oil refinery[43]. We exclude funding to the oil refinery[18].	Regional development
			80	Share of national co-financing to the EU JTF[18].	Regional development
**Slovakia**	Lower estimate	460	110	Transfer to coal mining company HBP to alleviate social and environmental consequences of coal mine closure. Confirmed in EU state aid cases.[44]	Coal mine closure
			250	EU transfer to support regions negatively affected by climate transition measures[10,45]. This includes one region that hosts coal industries, as well as regions that host chemicals and metals industries[10,45]. Since the exact flows allocated to each region and purpose are not specified, we assume that half of the overall EU funding is directed at the coal phase-out as the lower estimate[18,45].	Regional development
			100	Share of national co-financing to the EU JTF[18].	Regional development
	Upper estimate	340	240	Since the exact flows allocated to each region and purpose are not specified, we assume that the other half is also directed at the coal phase-out as the upper estimate[18,45].	Regional development
			100	Share of national co-financing to the EU JTF[18].	Regional development
**Slovenia**	Lower estimate	150	130	EU transfer to support regions negatively affected by climate transition measures[10,46]. This includes one region with an active coal industry, and one region with a former coal industry[10,46]. Since the exact flows and their purposes are not specified, we assume that half of the overall EU funding is directed at the coal phase-out as the lower estimate[18].	Regional development
			20	Share of national co-financing to the EU JTF[18].	Regional development

	Upper/ lower estimate	Total estimate ($_{2020}$ mln)	Estimate for each mechanism ($_{2020}$ mln)	Description of support (from original documents)	Type of support (coded)
	Upper estimate	170	140	Since the exact flows and their purposes are not specified, we assume that all EU funding is directed at the coal phase-out as the upper estimate[18,46].	Regional development
			30	Share of national co-financing to the EU JTF[18].	Regional development
**Spain**	Estimate	2,130	170	Transfer to regional governments to support environmental restoration of mining areas. Confirmed in national just transition plan. [47,48]	Regional development
			110	Transfer to municipalities to support building of digital, social and environmental infrastructures. Confirmed in national just transition plan.[48]	Regional development
			20	Transfer to coal workers for professional training and support in finding employment. Confirmed in national just transition plan.[48]	Unemployment support
			30	Transfer to the energy city foundation to support alternative energy infrastructure (e.g. hydrogen). Confirmed in national just transition plan.[48]	Renewables and low-carbon infrastructure
			30	Transfer to support business and small investment projects to generate employment in coal mining regions. Confirmed in national just transition plan.[48]	Regional development (SMEs)
			10	Transfers to local businesses affected by coal plant and mine closures. Confirmed in national just transition plan.[48]	Regional development (SMEs)
			120	Transfers to support economic recovery in coal mining regions. Confirmed in national just transition plan.[48]	Regional development (SMEs)
			360	Transfers to support renewable energy infrastructures in coal mining regions. Confirmed in national just transition plan.[48]	Renewables and low-carbon infrastructure
			890	EU transfer to support regions negatively affected by climate transition measures[10,49]. Since the original eligibility analysis and the final allocation news release only mention coal, we assume all EU JTF funding is paid in relation to the coal phase-out[18,49].	Regional development
			390	Share of national co-financing to the EU JTF[18].	Regional development
**Romania**	Estimate	1,550	500	Transfer to companies in the coal and renewables sectors to support alternative energy infrastructures. Pledge confirmed in Recovery and Resilience Facility.[50]	Renewables and low-carbon infrastructure
			890	EU transfer to support regions negatively affected by climate transition measures[10,51]. In total, the EU JTF benefits six Romanian regions, out of which two regions host active coal mining. Here, we include funding to these two regions[18,51].	Regional development
			160	Share of national co-financing to the EU JTF[18].	Regional development
**South Korea**	Lower estimate	10,940	10,940	Transfer to coal regions to support renewable energy infrastructure and create new jobs. Pledge confirmed in Korean New Deal to be paid from treasury.[52]	Renewables and low-carbon infrastructure
	Upper estimate	2,440	2,440	Transfer to coal regions to support renewable energy infrastructure and create new jobs. Additional to pledge from treasury.[52]	Renewables and low-carbon infrastructure

	Upper/ lower estimate	Total estimate ($\$_{2020}$ mln)	Estimate for each mechanism ($\$_{2020}$ mln)	Description of support (from original documents)	Type of support (coded)
**Vietnam**	Lower estimate	25,000	25,000	Own extrapolation of original pledge over the minimum duration of phase-out in line with the GCCP (2023-2040).International transfer to Vietnam to support coal phase-out and decarbonization in line with just transition. Different purposes are outlined, but it is not specified what share of funding will be allocated to each purpose: "facilitate investment into renewable energy and energy efficiency and to strengthen the electricity grid in Viet Nam […], training, retraining and vocational support for employment; ensure better living conditions for workers after the transition […],help meet the needs of those most affected by the green transition, such as workers and communities in sectors and areas affected by the transition […], negotiate - with the support of partners - the halting of investment in coal-fired power plants to deliver these goals, where appropriate; negotiate the closure of old, inefficient unabated coal-fired power plants to facilitate access to clean energy […], create an enabling environment for businesses to proactively participate in the transformation process […],design mechanisms to assist ensuring affordable electricity for affected, vulnerable and low-income groups […]". Pledge confirmed in international agreement.[53]	JETP to national government *Coal power plant closure, renewables and low-carbon infrastructure, unemployment support, regional development*
	Upper estimate	62,930	62,930	Own extrapolation of original pledge over the maximum duration of phase-out in line with the GCCP (2023-2049). (Methods, Supplementary Note 1).	JETP to national government *Coal power plant closure, renewables and low-carbon infrastructure, unemployment support, regional development*

Supplementary Table 2. Description five types of support identified in coal phase-out compensation policies.

Type of support	Description of what this entails
Regional development (support to regions)	Funding aimed at improving infrastructure and quality of life in coal regions. This can include funding for IT-infrastructure, new roads, railroads, funding for research facilities, etc.<br>NOTE: We include the EU just transition fund under "Regional development". However, individual flows may be used by regions to support the local economy, which may ultimately support workers, or to increase local renewables capacity.
Regional development (support to SMEs)	Funding aimed at diversification of the local economy, to small and medium-sized enterprises (SMEs) other than energy companies in coal regions.
Coal power plant and mine closure (support to companies)	Funding that is aimed at the closure of coal-fired power plants or coal mines. This can entail compensation to companies for foregone revenues, financing for mine restoration, or financial support for companies to invest in technologies other than coal.
Renewables and low-carbon infrastructure (support to companies and regional governments)	Funding that is aimed at supporting the deployment of renewables, improvements to the electricity grid or additional infrastructure to be able to support increased renewables and low-carbon capacity. This can include support for renewables companies or transfers to regions to invest in renewables capacity and low-carbon infrastructure.
Unemployment support (support to workers and regional governments)	Funding that is aimed at easing the transition for coal workers. This can include transfers directly to coal workers to compensate for the job loss or funding to support re-training facilities and job agencies.

Supplementary Table 3. Overview of countries with coal phase-out pledges.

Data on coal phase-out pledges is based on Vinichenko et al.[1] Countries which have no coal capacity at the time of making the phase-out pledge and countries which don't have a year associated with their coal phase-out pledge are excluded from our analysis, and shown here in the last two rows. Avoided emissions for countries with coal phase-out pledges have been calculated vis-a-visa a reference scenario under which all operating and planned power plants are retired at a nationally-specific average retirement age (Methods). *****Countries which are included in the EU Just Transition Fund, but where there is no evidence of just transition compensation for coal, or of national just transition plan or policy in relation to coal. See Supplementary Table 1 for compensation estimates and sources.

Country	Year of phase-out pledge	Type of pledge	Current (and previous) phase-out pledge	GWe & (Number) of operating coal plant units	Avoided emissions (Mt CO2)	Pledged compensation (USD bln)
Countries with coal phase-out pledge and compensation policy						
Germany	2019	PPCA	2030 (2035-2038)	43 (172)	1489 (1207-1770)	66 (66-67)
Viet Nam	2021	GCCP	2040s	19.7 (64)	542 (191-1234)	43 (25-63)
South Korea	2021	GCCP	2050	35.7 (104)	540	12.2 (11-13)
Indonesia	2021	GCCP	2040s	34 (268)	509 (179-1227)	55 (31-79)
Italy	2017	PPCA	2025	10.5 (34)	470	1.1 (0.9-1.3)
Czechia	2022	National	2033	10 (182)	447	1.7 (1.2-2.2)
Poland	2021	GCCP	2049	31.3 (292)	435	15.3
Chile	2021	PPCA	2040 or earlier	5 (25)	390 (263-518)	NA
Netherlands	2017	PPCA	2029 (2030)	4.8 (6)	387	0.18 (0.06-0.29)
Canada	2017	PPCA	2030	9.1 (33)	156	1.2
Greece	2019	PPCA	2028 (2023)	4.6 (16)	148 (121-176)	1.8 (1.2-2.3)
Slovenia	2021	PPCA	2033	0.7 (6)	80	0.24 (0.16-0.32)
France	2017	PPCA	2022	3.3 (51)	73	0.1
Bulgaria	2021	EU	2038 or earlier	4.6 (38)	50 (45-55)	1 (0.26-1.74)
Ukraine	2021	PPCA	2040 or earlier	24.7 (125)	44 (36-52)	NA
North Macedonia	2021	PPCA	2030	0.7 (3)	30	NA
Slovakia	2019	PPCA	2025	1.1 (32)	28	0.6 (0.5-0.8)
Portugal	2017	PPCA	2021 (2030)	1.9 (6)	26	0.3
Spain	2021	PPCA	2030 or earlier	3.1 (14)	25	2.1
Finland	2017	PPCA	mid-2029 (2030)	2.6 (20)	17	0.28
Croatia	2021	PPCA	2033 or earlier	0.2 (3)	15	0.17 (0.11-0.22)
Romania	2021	EU	2032	5.5 (29)	9	1.6
Hungary	2021	PPCA	2029	1.2 (13)	0.1	0.3
Total	-	-	-	**260 (1540)**	**5930 (4900-7790)**	**204 (159-253)**
Countries with coal phase-out pledge and no compensation policy						
Israel	2018	PPCA	2025 (2030)	4.9 (13)	169	
Philippines	2021	GCCP	2040s	10.6 (64)	158 (0-376)	
Kazakhstan	2021	GCCP	2050	12.2 (124)	109	
United Kingdom	2017	PPCA	2024 (2025)	15.3 (45)	45	
Panama	2021	National	2023	0.4 (5)	45	
Ireland*	2018	PPCA	2025	0.9 (3)	30	
Botswana	2021	GCCP	2040s	0.8 (9)	25 (2-36)	
Mauritius	2021	PPCA	2030	0.2 (5)	22	
Denmark*	2017	PPCA	2028-2030 (2030)	2.5 (9)	18	
Senegal	2018	PPCA	2040s	0.2 (3)	18	
Zambia	2021	GCCP	2040s	0.3 (3)	16 (4-22)	
Sri Lanka	2021	GCCP	2040s	0.9 (3)	13 (0-41)	
Peru	2020	PPCA	2022	0.1 (1)	10	
Sweden*	2017	PPCA	2020 (2022)	0.1 (3)	8	
Brunei Darussalam	2021	GCCP	2040s	0.2 (4)	5 (0-9)	
Austria*	2017	PPCA	2020 (2022)	0.8 (9)	4	
New Zealand	2017	PPCA	2030	0.5 (9)	0.1	
Montenegro	2021	PPCA	2035	0.2 (1)	0	
Myanmar	2021	NDC	2050	0.2 (5)	0	
Singapore	2021	PPCA	2050	0.1 (2)	0	

Country	Year of phase-out pledge	Type of pledge	Current (and previous) phase-out pledge	GWe & (Number) of operating coal plant units	Avoided emissions (Mt CO2)	Pledged compensation (USD bln)
Total	-	-	-	50 (320)	680 (470-940)	-

Countries with coal phase-out pledge but no coal power: Albania, Agola, Azerbaijan, Belgium, Costa Rica, Côte d'Ivoire, Cyprus, El Salvador, Egypt, Estonia, Ethiopia, Fiji, Iceland, Latvia, Lithuania, Lichtenstein, Luxembourg, Maldives, Marshall Islands, Mauritania, Niue, Nepal, Norway, Somalia, Switzerland, Tuvalu, Uruguay, Vanuatu

Countries which are PPCA members but do not have a date associated with their coal phase-out pledge: Dominican Republic, Kosovo, Mexico, United States

Supplementary Table 4. Share of domestic and international finance separated by separated by recipients of ODA-finance, recpients of EU-fiannce, and donor countries of international finance.

EU and ODA donor countries include all countries with coal phase-out pledges and compensation policies that are ODA donors, and are also net-donors of EU finance. EU recipient countries are EU countries with coal phase-out pledges and compensation policies that are not ODA recpients, but are net-recipients of EU finance. International finance for these two groups essentially includes EU-transfers form the EU Just Transition Fund and Recovery and Resilience Fund. ODA recipient countries include all countries with coal phase-out pledges and compensation policies that are ODA recipients. This essentially includes the three JETP countries, South Africa, Vietnam and Indonesia, and international finance to these countries includes JETP-funding. Supplementary Table 5 shows which countries are included in each group.

	Share of domestic compensation	Share of international compensation<br>(paid by EU, or JETP-donors)
ODA-recipient countries	0%<br>(0-0)	87%<br>(82-90)
EU-recipient countries	15%<br>(15-15)	9%<br>(7-12)
EU+ODA donor countries	85%<br>(85-85)	4%<br>(3-11)
**Total**	**100%**	**100%**

Supplementary Table 5. EU/ODA donors and recipients.

Countries eligible to receive ODA-funding retrieved from the OECD[54]. Countries receiving EU funding retrieved from a report on net donors and recipients of EU funding – donors pay more money into the EU budget than they receive in funds[55]. If a country is a recipient in one of the two columns, it is considered an overall recipient of international finance.

Country	ODA recipient or donor	EU recipient or donor
**Countries with coal phase-out pledge and compensation policy**		
Germany	Donor	Donor
Viet Nam	Recipient	-
South Korea	Donor	-
Indonesia	Recipient	-
Italy	Donor	Donor
Czechia	Donor	Donor
Poland	Donor	Recipient
Netherlands	Donor	Donor
Greece	Donor	Recipient
Canada	Donor	-
Slovenia	Donor	Recipient
France	Donor	Donor
Bulgaria	Donor	Recipient
Slovakia	Donor	Recipient
Finland	Donor	Donor
Portugal	Donor	Recipient
Spain	Donor	Recipient
Croatia	Donor	Recipient
Romania	Donor	Recipient
Hungary	Donor	Recipient
**Countries with coal phase-out pledge but no compensation policy**		
Israel	Donor	-
Philippines	Recipient	-
Kazakhstan	Recipient	-
United Kingdom	Donor	-
Panama	Recipient	-
Ireland	Donor	Donor
Mauritius	Recipient	-
Denmark	Donor	Donor
Sri Lanka	Recipient	-
Peru	Recipient	-
Sweden	Donor	Donor
Austria	Donor	Donor
Zambia	Recipient	-
New Zealand	Donor	-
Montenegro	Recipient	-
Botswana	Recipient	-
Myanmar	Recipient	-
Senegal	Recipient	-
Singapore	Donor	-

Supplementary Table 6. Construction cost of new coal plants.

Data on coal power plants built in European countries between 2010 and 2022 from the S&P World Electric Power Plants database[56]. Data on the cost of building these power plants is retrieved through a systematic google search (see Methods). Currencies differ depending on the respective source. Values are converted to USD using the average annual exchange rate for the year in which each report was published, retrieved from the OECD[57].

Country	Name of power plant (Year of construction)	MW capacity	Cost (USD2020 bln)
Bulgaria	AES Galabovo (2011)	670	USD 1.7bn[58]
Czechia	Prunerov (2016)	750	USD 1.3bn[59]
	Kladno (2014)	135	NA
	Ledvice (2020)	660	USD 2.3bn[60]
Germany	Boxberg (2012)	675	USD 1bn[61]
	Datteln (2020)	1100	USD 1.7bn[62]
	GKM (2015)	911	USD 1.7bn[63]
	Lunen-4 (2013)	800	USD 1.9bn[64]
	Moorburg (2015)	1730	USD 3.3bn[65]
	Neurath (2012)	2240	USD 4.7bn[66]
	RDK (Karlsruhe) (2014)	912	USD 2bn[67]
	Walsum-10 (2013)	790	USD 1.5bn[68]
	Westfalen (2014)	820	USD 1.1bn[69]
	Wilhelmshaven-C (2015)	800	USD 1.8bn[70]
Italy	Torrevaldaliga Nord (2010)	1320	NA
Netherlands	Centrale Rotterdam (2014)	800	NA
	Eemshaven (2015)	1600	USD 3.4bn[71]
	Maasvlakte (2016)	1116	USD 1.9bn[72]
Poland	Belchatow (2011)	858	USD 2.4bn[73]
	Jawrozno-III (2020)	910	USD 1.8bn[74]
	Opole (2019)	1800	USD 3.6bn[75]
	Turow (2021)	496	USD 1.1bn[76]
Slovenia	Sostanj 6 (2015)	600	USD 1.6bn[77]

Supplementary Table 7. Cumulative and annual compensation compared to other international funding.

Cumulative and annual compensation are reported as those indicated or implied by compensation policies. Values directly derived from primary and secondary sources are indicated in bold and those which are calculated as implied by compensation policies are marked in italics. Sources on domestic and EU funding generally report cumulative coal phase-out compensation while JETPs report annual coal phase-out compensation. Other forms of international funding are all general reported in primary and secondary sources as annual, with the exception of the Indian prime minister's request for climate financing. See column Notes for details on the calculations of compensation implied by coal phase-out compensation policies.

			Compensation amount		Notes
			Cumulative ($billion)	Annual ($billion/yr)	
**Compensation policies**	Domestic funding		92 (89-95)	*6.4 (4.7-8)*	Cumulative value from primary and secondary sources (Supplementary Table 1). Annual amount calculated by dividing the number of years between the year when the coal phase-out pledge was made and the pledged phase-out date.
	EU funding (JTF and RRF)		14 (13-16)	*1.1 (1-1.7)*	
	JETP funding	South Africa	8.5	2.3 (1.7-2.8)	Annual value from JETPs[78]. Lower estimate of cumulative amount calculated by extrapolating pledged JETP support to the earliest pledged coal phase-out date (2040) under the GCCP. Upper estimate of cumulative amount calculated by extrapolating pledged JETP support to the latest pledged coal phase-out date (2049) under the GCCP. Central estimate calculated by extrapolating pledged JETP support to the central pledged coal phase-out date (2045) under the GCCP (see also Supplementary Note 1). Cumulative amount for South Africa is the total amount currently pledged in its JETP. Extrapolation for an upper estimate is not possible for South Africa which does not have an explicit coal phase-out pledge.
		Vietnam	*43 (25-63)*	2.1 (1.5-2.4)	
		Indonesia	*55 (31-79)*	2.7 (1.8-3)	
**Projected compensation for India and China in line with climate targets**	1.5°C	China	*2,200 (1,200-5,300)*	*156 (125-522)*	Central cumulative amount calculated by applying average compensation per ton CO_2 to median avoided emissions under IPCC AR6 pathways for the respective temperature trajectory; uncertainty range calculated with top models across our five regression analyses; their confidence intervals, and IQR of avoided emissions under IPCC pathways. Annual amount calculated by dividing total compensation by the amount of years between 2022 and the median and IQR coal phase-out dates for IPCC pathways respectively (Methods).
		India	*1,000 (500-1,600)*	*66 (37-90)*	
	2°C	China	*1,600 (1,000-4,800)*	*91 (80-300)*	
		India	*800 (500-1,300)*	*39 (23-58)*	
	2.5°C	China	*1,100 (900-2,700)*	*41 (33-120)*	
		India	*500 (300-1,000)*	*15 (8-26)*	
**Existing and planned international and domestic financial flows**	Gross ODA	Indonesia	*59 (47-69)*	2.5 (1.5-3.4)	Annual gross ODA from OECD.[5] Cumulative gross ODA for Indonesia and Vietnam calculated by multiplying ODA flows by the range of coal phase-out pledges under the GCCP (2040-2049) and calculating the mean for the central estimate (see also Supplementary Note 1). Extrapolation not possible for South Africa because it does not have a coal phase-out pledge. For China and India, cumulative gross ODA is
		Vietnam	*81* (64-95)	3.4 (2.2-4.9)	
		South Africa	NA	1.4 (1.1-1.7)	
		India	*107 (85-143)*	5.5 (4.2-6.5)	
		China	*24*	1.5	

		(16-31)	(1.2-1.8)	extrapolated over the number of years between 2022 and median and IQR the coal phase-out dates under all 1.5°C- and 2°C-compatible IPCC pathways. We report total global ODA for the year 2021, retrieved from the OECD.
	Total global ODA	-	176	
Coal production subsidies	India	*2* *(2-3)*	0.1 (0.08-0.2)	Annual coal production subsidies from OECD[2]. Cumulative subsidies are calculated as the most recent subsidy data multiplied by the number of years between 2022 and median and IQR the coal phase-out dates under IPCC pathways respectively.
	China	*16* *(8-24)*	1.1 (0.01-2)	
COP26 Climate finance request	India	1,000	*56.1* *(45.5-66.7)*	Cumulative value is climate finance of request $1 trillion by India's prime minister at COP26.[6] Annual value is calculated by dividing the request by the median and IQR duration of coal phase-out in line with 1.5°C and 2°C consistent pathways (see Methods).
COP Climate finance pledge		*1,450* *(700-2,200)*	100	Annual COP Climate finance pledge to all developing countries from OECD[7]. Cumulative amount is calculated by extrapolating the pledge ($100 billion per year) over the number of years between 2022 and median and IQR the coal phase-out dates under IPCC pathways respectively.

Supplementary Table 8. Difference in avoided emissions and compensation estimates between pathway categories.

Avoided emissions and compensation estimates based on individual AR6 pathway categories. Uncertainty ranges indicate uncertainties from distribution of IPCC pathways within each category.

Region	Avoided emissions estimates from 1.5°C consistent pathway categories			Avoided emissions estimates from 2°C consistent pathway categories		
	C1	C2	C1&C2 combined	C3	C4	C3&C4 combined
India+	27 Gt (25-29)	24 Gt (17-31)	26 Gt (21-30)	22 Gt (18-26)	19 Gt (16-25)	21 Gt (17-25)
China+	68 Gt (60-71)	49 Gt (37-66)	60 Gt (42-69)	50 Gt (37-60)	38 Gt (27-48)	43 Gt (35-57)

Region	Compensation estimates from 1.5°C consistent pathway categories			Compensation estimates from 2°C consistent pathway categories		
	C1	C2	C1&C2 combined	C3	C4	C3&C4 combined
India+	$1 trn (0.6-1.6)	$0.9 trn (0.4-1.6)	$1 trn (0.4-1.6)	$0.8 trn (0.5-1.4)	$0.7 trn (0.4-1.3)	$0.8 trn (0.5-1.3)
China+	$2.5 trn ($2-5.3)	$1.8 trn (1.2-5.2)	$2.2 trn (1.2-5.3)	$1.9 trn (1.2-5)	$1.4 trn (0.9-4.4)	$1.6 trn (1.2-4.8)

Supplementary Table 9. Bottom-up estimates of 'real' coal phase-out costs compared to estimates in this paper.

Source	Estimate for…	Coal phase-out date for analysis	Estimated amount
**iForest report[79]**	…'real' cost of coal phase-out by 2050 in India (bottom-up estimate)	2050	$1 trn
**This paper (see Table 4 in main text)**	…cost of coal phase-out in India in line with 1.5°C- and 2°C-compatible IPCC AR6 pathways under extrapolation of existing compensation policies (top-down estimate)	1.5°C -compatible central estimate: 2038 (range 2035-2041)	1.5°C-compatible central estimate: $1 trln (range 0.5-1.6)
		2°C -compatible central estimate: 2045 (range 2040-2045)	2°C-compatible central estimate: $0.8 trn (range 0.5-1.3)

Supplementary Notes

Supplementary Note 1. Just Energy Transition Partnerships

Just Energy Transition Partnerships (JETPs) are a new mechanism to support coal-dependent countries in decarbonisation and climate mitigation[80,81]. The first JETP was established at COP26 between South Africa as recipient and France, Germany, the United Kingdom, the United States, and the European Union as donors[78,82]. Since then, JETPs have also been established with Vietnam, Indonesia and Senegal as recipients, and the group of donor countries has expanded (and now known as the International Partners Group and also includes Denmark, Italy, Canada, Japan and Norway[35,53]). A Just Energy Transition Partnership is also in discussion with India[81,83].

With the exception of the JETP with Senegal (see Methods), each JETP pledges a certain level of funding (currently between USD7 bn - USD20 bln) over three to five years along with administrative and technical assistance for coal phase-out[35,53,78,84]. Usually, half of the funds are provided by donor countries, and the other half is mobilized from private finance, for example from the "Glasgow Financial Alliance for Net Zero" - a working group of several large banks (including Bank of America, Deutsche Bank, and HSBC)[35,53,78]. In our analysis, we only include compensation funded by donor countries (see Methods). There has been criticism of the public funding portion under JETPs from recipient countries since the largest share of the funding seems to be planned in the form of loans rather than grants, which some recipient countries are concerned may negatively affect the debt-to-GDP ratio[85] and decrease the overall value of JETP support. Others, however, argue that JETP funding is likely to have only small, and possible positive, impacts on countries' credit profiles[86].

Under the agreements, recipient countries lead the development of a transition plan, to be endorsed by donor countries and to ensure a constructive use of funds[81]. If the plan is endorsed by donor countries and then followed by recipient countries, the JETPs provide opportunities for more support for decarbonisation in the longer term[35,53,78]. Given that the vision of the JETPs is to be renewed and extended, we establish a plausible estimate of compensation over the entire duration of coal phase-out in Indonesia and Vietnam by extrapolating currently-pledged compensation until the earliest, central and latest possible date of coal phase-out (2040, 2045, and 2049) for Indonesia and Vietnam (Methods). For South Africa, such an extrapolation is not possible because there is no phase-out date specified. The central estimate we report is the average between already-committed funding and our upper estimate.

The JETPs also include estimates of emission reductions compared to the countries' current pathways to be achieved through the partnerships. The JETP for Vietnam envisions a "cumulative reduction of around 200 megatons of greenhouse gas emissions by 2030, and a further 300 megatons by 2035"[53] – roughly comparable to our central estimate of avoided emissions of coal phase-out (Supplementary Table 3). The JETP for Indonesia foresees a "cumulative reduction of more than 300 megatons in greenhouse gas emissions through 2030 and a reduction of well above 2 gigatons through 2060 from Indonesia's current trajectory"[35] which is roughly comparable to our esimates in the near-term and somewhat higher than them in the longer term (Supplementary Table 3). The JETP for South Africa "is expected to prevent

up to 1-1.5 gigatons of emissions over the next 20 years"[78] though so far there is no definite coal phase-out pledge or plan.

Supplementary Note 2. Uncertainty and robustness analysis

We conduct an uncertainty analysis of our findings testing for the effect of: parametric uncertainties arising from coal phase-out pledges and compensation policies; model uncertainties arising from using different control variables in different models and the confidence intervals of specific models; and pathway uncertainties arising from different coal phase-out trajectories for China and India envisioned in different mitigation pathways and leading to different levels of avoided emissions. We test the effect of these uncertainties on three aspects of our analysis:

1. our finding that compensation is proportional to coal phase-out ambition;
2. our assessment of the amount of compensation per unit of avoided emissions ($/tCO2); and
3. our compensation estimates for China and India.

We first describe how we quantify each uncertainty and then how we test their effect on different aspects of our analysis.

Types of uncertainties

For **parametric** uncertainties, we test the effect of uncertainties in both coal phase-out pledges and compensation policies. We parameterize the first uncertainty using the range in timing of the coal phase-out pledge for all national cases where the pledged coal phase-out date has shifted overtime or in the case of Indonesia and Vietnam, where the coal phase-out pledges do not contain a single date (Supplementary Table 3). The former captures cases like Germany, which had originally pledged to phase out coal latest by 2038, and has later pledged to phase out coal by 2030[87,88]. In this case, the optimistic phase-out date is 2030, and the pessimistic phase out date is 2038. The central estimate is the mean of the optimistic and pessimistic phase-out. In the case of Indonesia and Vietnam, the countries have pledged to phase out coal power "in the 2040s"[89]; in these cases we use 2040 as the optimistic phase-out date, 2045 as the central phase-out date, and 2049 as the pessimistic phase-out date.

The second parametric uncertainty we test arises from uncertainties associated with the compensation policies themselves (Supplementary Table 1 and 3, Methods). The largest uncertainty in compensation policies is in the case of Indonesia and Vietnam. In these two cases the JETPs specify total funding for a period of three to five years with expectations for "continuation of the partnership"[35,53]. To estimate a plausible uncertainty range in compensation for these two countries, we first calculate a range of *annual* compensation for each country by dividing the total committed compensation by three, four or five years and then extrapolating the range in annual amounts to the latest, central and earliest possible phase-out dates (Supplementary Table 3, Table 2, Methods):

$$(1)\ compensation_{JETP\ country_{upper}} = \frac{JETP\ funding_{JETP\ country}}{3\ years} * (2049 - 2023)$$

$$(2)\ compensation_{JETP\ country_{central}} = \frac{JETP\ funding_{JETP\ country}}{4\ years} * (2045 - 2023)$$

$$(3)\ compensation_{JETP\ country_{lower}} = \frac{JETP\ funding_{JETP\ country}}{5\ years} * (2040 - 2023)$$

The uncertainty in compensation policies also captures cases where we could not confirm the compensation payments in official documentation. This captures for example cases like Greece, where we could not identify one of the individual compensation payments in an official government documentation, and we thus capture this payment as part of the higher estimate. The lower estimate contains only those payments which we could confirm in official government documentation, and the central estimate is the mean of higher and lower estimate.

For **model uncertainties,** we test the effect of uncertainties arising from the effect of using different control variables and the models' confidence intervals (Supplementary Note 3, Methods, Supplementary Tables 12-16).

Finally, for **IPCC pathway uncertinaties** we test the effect of uncertainties arising from the variation of the speed of coal phase-out under different IPCC pathways, leading to a range of avoided emissions estimates for China+ and India+ under each temperature trajectory (Table 3). We test for this uncertainty using the IQR of avoided emissions for each temperature category (Methods).

Uncertainty analysis of the relationship between compensation and avoided emissions

We first conduct an uncertainty analysis to ensure that our finding that the level of compensation is proportional to the level of coal phase-out ambition is robust to the two types of parametric uncertainties; and to the model uncertainty of controlling for different combinations of variables capturing characteristics of the coal sector and the national context. We do this by conducting five iterations of our regression analysis (Supplementary Note 3, Methods): a central set of regression models using both the central compensation estimate and central pledge estimates (Supplementary Table 12) and four sensitivities where we vary either the pledge estimates (pessimistic and optimistic) (Supplementary Tables 13-14), or the compensation estimates (lower and higher) (Supplementary Tables 15-16). We find that avoided emissions consistently is the best predictor of compensation – significant at the highest level across our ten best performing models (ranked by AIC) across all five iterations of our regression analysis. This shows that the relationship between the level of compensation and the level of coal phase-out ambition is robust to both parametric uncertainties and to different combinations of control variables.

Uncertainty analysis of the compensation per ton of avoided CO_2 emissions

Second, we test whether our assessment of the amount of compensation per unit of avoided emissions ($/tCO2) is robust to parametric and model uncertainties.

We test parametric uncertainties using two different methods: calculating the average of compensation per ton avoided emissions and our regression analysis. For the first, we calculate the average of compensation per ton avoided emissions by using both a pessimistic and optimistic interpretation of the pledges along with a central estimate for compensation and a lower and higher estimate for compensation and central estimate for pledges (Supplementary Table 10). We find that the two parametric uncertainties (range of pledges and compensation estimates) have similar effects on the average, which overall varies roughly between \$29-\$46/tCO2.

We also identify the range of coefficients for compensation per ton of avoided CO_2 in our top performing model using both the central compensation estimate and pledge interpretation and within each sensitivity run with the same control variables. The top model in the central regression run controls for coal mining and whether a country is a net-recipient of international funding. For pledge ambition, this corresponds to M8 in the pessimistic interpretation of the pledges and M5 in an optimistic interpretation of the pledges; for compensation policy this corresponds to M3 for the lower compensation estimate and M9 for the higher compensation estimate. Using the regression method to estimate uncertainties results in a narrower range of compensation per ton of avoided CO_2 emissions. This is not surprising because the regression analysis accounts for variation in national contexts and coal sectors.

We next test the effect of model uncertainty with the range of compensation per ton avoided CO_2 emissions across the top ten models using our central compensation parameters and with the confidence intervals of the compensation per ton of avoided CO_2 emissions using the top model under the central parameters (Supplementary Table 10). Both methods result in a similar range and that model uncertainties are smaller than parametric uncertainites.

Supplementary Table 10. Effect of parametric and model uncertainties on the amount of compensation for one ton of avoided emissions (\$/ton avoided CO_2 emissions).

Parameteric uncertainties cover both uncertainties in pledge ambition and compensation policies and are reported using the average calculation and the co-efficient of compensation per ton avoided CO_2 emissions across relevant regression sensitivities (Methods). For the average method, the uncertainty is calculated using the range of pledge ambition and compensation policies together with the central estimate for the other (i.e. optimistic and pessimistic pledge interpretation and central compensation estimate for compensation; upper and lower compensation estimates with central pledge interpretation). The effect of parametric uncertainties is also reported using the coefficient for ton of avoided CO_2 emissions in the top model from the central regression run along with the model within each sensitivity model with the same control variables (Supplementary Tables 12-16). Model uncertainty is captured using the central regression run and the range of coefficients for compensation per ton avoided emissions CO_2 across the top-ten models as well as the confidence interval for compensation per ton avoided CO_2 emissions in the top model.

Parametric uncertainty		
	average compensation/tCO$_2$	*regression method*
pledge ambition	37.5 [28.5-45.9]	39.8 [33.3-41.0]
compensation policy	37.5 [28.9-46.0]	39.8 [37.8-42.4]
**Model uncertainty**		
top ten models (central parameters)	-	39.8 [34.0-42.2]
confidence intervals top model (central paramters)	-	39.8 [36.0-43.6]

We also compare the compensation per ton of avoided emissions calculated using the average across all countries (weighted by avoided emissions) and across the five sets of regression models (Supplementary Tables 12-16). We find good agreement between these two methods:

The range of average is $28.5-45.9 and the range across regression models is $27.3-45.0. The consistency between these two methods confirms the robustness of our approach.

Uncertainty analysis of compensation estimates for China and India

Finally, we estimate the uncertainty of compensation estimates for China and India to IPCC pathway uncertainties, parametric uncertainties, and model uncertainties using two methods – the average method and the regression method. In both cases, we use the IQR of avoided emissions associated with 1.5°C- ,2°C- and 2.5°C degree -compatible AR6 pathways.

For the "average compensation/tCO2", we calculate a lower estimate using the Q1 level of avoided emissions in IPCC pathways along with the lowest average of compensation per-ton of avoided CO2 emission (Supplementary Table 10); we calculate an upper estimate using the Q3 level of avoided emissions in IPCC pathways along with the highest average of compensation per-ton of avoided CO2 emission (Supplementary Table 10).

For the "regression method", we use the top ten models each of our five sets of regression analyses along with the range of confidence intervals for each variable each model (Supplementary Tables 12-16). For the lower estimate, we the confidence intervals from each coefficient and use the Q1 level of avoided emissions in IPCC pathways, for the upper estimate, we add the confidence intervals to each coefficient and use the Q3 level of avoided emissions in IPCC pathways.

Supplementary Table 11 show our results for China+ and India+ respectively. In general, the estimates based on averages tend to be lower than the estimates based on the regression analysis, indicating that variables related to the strength of the coal sector and national capacity also affect the amount of compensation. Overall, compensation estimates remain in the same order of magnitude in both methods and across our uncertainty tests, indicating their robustness to the three types of uncertainties we test here. Additionally, we find that the uncertainty range is larger above our central estimate than below.

Supplementary Table 11. Uncertainty ranges for China based on different types of uncertainties.

Three types of uncertainty ranges for compensation estimates for China+: The first is based on parametric uncertainty, calculated using the average of compensation per ton of avoided emissions with (1) a range of existing pledge ambition, and (2) a range of compensation estimates (see Supplementary Table 3). The second is based on uncertainty from regression models, calculated using (1) the top ten models (M1-10) with central parameter estimates, (2) the confidence interval of the top model (M1) with central parameter estimates, and (3) the confidence intervals of the top ten models with central parameter estimates (see Supplementary Table 11). The third is based on the range of avoided emissions under IPCC pathways, calculated using the interquartile range of avoided emissions estimates across IPCC pathways multiplied by average of compensation per ton avoided emissions based on central parameter estimates.

Uncertainty range..	1.5°C	2°C	2.5°C
For China…			
IPCC pathway uncertainty (IQR of avoided emission in IPCC pathways)	42-69 Gt	36-57 Gt	23-37Gt
average compensation/tCO_2 ($ trillion using range of averages)	$1.2-3.2 trn	$1-2.6 trn	$0.9-1.7 trn
regression method ($ trillion using top ten models across five sets of regression runs and range of confidence intervals)	$1.4-5.3 trn	$1.2-4.8 trn	$0.8-4 trn
Full uncertainty range China	$1.2-5.3 trn	$1-4.8 trn	$0.8-4 trn
For India…			
IPCC pathway uncertainty (IQR of avoided emission in IPCC pathways)	21-30 Gt	17-25 Gt	8-18 Gt
average compensation/tCO_2 ($ trillion using range of averages)	$0.6-1.4 trn	$0.6-1.2trn	$0.4-0.8 trn
regression method ($ trillion using top ten models across five sets of regression runs and range of confidence intervals)	$0.5-1.6 trn	$0.5-1.3 trn	$0.3-1 trn
Full uncertainty range India	$0.5-1.6 trn	$0.5-1.3 trn	$0.3-1 trn

Supplementary Note 3. Regression analysis

We conduct a multivariable regression analysis in order to test our hypothesis that the level of ambition of coal phase-out is comparable to the amount of compensation. Our sample consists of all countries with coal phase-out pledges, and for which all of our independent variables were available (Methods). Our outcome variable is our estimate of coal phase-out compensation. To test the robustness of our hypothesis to uncertainties in the empirical compensation estimates (Methods, Supplementary Note 2, Supplementary Table 3), we conduct several iterations of our regression analysis where we vary our outcome variable using our lower, central, and higher compensation estimates for one iteration respectively. In our regression analysis, we test for the effect of the ambition of coal phase-out while controlling for variables capturing additional mechanisms which are likely to affect the amount of compensation paid.

These mechanisms fall broadly into two groups: characteristics of the coal sector affecting likely resistance against coal phase-out, and characteristics of the national context affecting governments' capacities to overcome this resistance. We identify the individual mechanisms based on evidence from previous literature on enablers and barriers of coal phase-out in different contexts[1,90–93]. Our analysis contributes to this literature by further examining the relationship between compensation policies and the ambition of coal phase-out pledges across contexts, thus advancing the understanding of the policy effort needed to phase-out coal in difficult contexts.

Below, we first describe the individual mechanisms we identify from previous literature which are likely to affect coal phase-out and the variables we choose that capture these mechanisms. We then describe our regression analysis and our results in detail.

Selection of dependent variables and mechanisms

Compensation is paid to those actors that are negatively affected by a coal phase-out pledge to address resistance and enhance the feasibility of coal phase-out as well as to make transitions more just and equitable[94–96].

The level of **ambition of the coal phase-out pledge** is likely to affect the level of resistance, which can arise due to the premature retirement of infrastructure such as power plants. Premature retirement leads to stranded capacity [97,98], for which investors and companies would otherwise have retained further profits, or workers would have retained their jobs. Additionally, pledging faster coal phase-out is likely to be more difficult since companies and workers need to more quickly adjust to the phase-out.

Characteristics of the coal sector are likely to affect this resistance in the national context. For example, resistance is likely to be stronger in the case of a **stronger coal sector**[91]. In particular, coal sectors are likely to be stronger and resistance higher in countries with domestic coal mining **mining** in a country as evidenced by the fact that in several countries, such as Spain, Poland and Germany, workers and companies associated with the coal mining industry protested against coal phase-out[99–101] . It may also be stronger in cases where the **coal power sector is concentrated** in certain regions, as opposed to being spread out over the

entire country, since the regional government and economy in these cases bear a large share of the cost[96,102].

Characteristics of the national context are also likely to affect the amount of compensation paid particularly those characteristics which affect the capacity of the government to overcome resistance to coal phase-out. On the one hand, **state capacity** captures the extent to which governments are able to formulate and implement certain policies. Governments with higher capacity to formulate policies, and oversee their implementation, are likely to mobilise higher amounts of compensation for coal phase-out. Additionally, governments with higher **economic capacity** are likely to be able to bear the costs of policy measures and in the case of coal-phase out to mobilize funds for compensation policies. In addition to states' own economic capacity, the availability of **international funding** may increase the funding available for coal phase-out. Such funding may be available through different institutions – the EU allocates funding to its member states for regional development and climate mitigation, and official development assistance (ODA) may be allocated towards climate mitigation efforts as well.

We identify at least one variable for each of these mechanisms:

Variables related to the ambition of national coal phase-out:

- **Higher avoided emissions** of the coal phase-out is expected to be associated with **higher** compensation because it indicates that younger coal power plants are retired and more stranded assets which is likely to be associated with resistance to coal phase-out. To test our hypothesis to uncertainties in empirical coal phase-out ambition, we conduct three iterations of regression analyses where we vary our avoided emissions estimates using pessimistic, central and optimistic coal phase-out pledges respectively, while keeping our outcome variable stable.
- **Shorter timespan over which coal is phased out** is expected to be associated with **higher** compensation, because it indicates that coal power is phased out faster, which may lead to more resistance from both the coal industry and regions where the coal industry is located which need to quickly adapt.

Control variables related to the characteristics of the coal sector:

- Strength of the coal sector:
  - A **large coal power plant fleet** is expected to be associated with **higher** compensation cost due to more infrastructure needing to be retired, and the resistance of actors dependent on this infrastructure (plant workers and owners, and regions where the coal industry is located).
  - **Higher generation of coal power** is expected to be associated with **higher** compensation, since it is associated with the strength of the coal industry and replacing more coal power requires more (financial) effort.
  - **Higher levels of domestic coal mining** is expected to be associated with **higher** compensation, because domestic coal is important for energy security and is likely to be associated with resistance from actors dependent on this industry (mining workers and companies as well as regions rich in coal reserves).

- Larger numbers of coal workers are expected to be associated with higher compensation because more coal workers may indicate more resistance, as well as more potentially unemployed workers to be integrated into the labor market.
- Regional concentration of coal power capacity:
  - Higher regional concentration of coal power plant fleet is expected to be associated with higher compensation cost due to the high distributional cost imposed on certain regions, which is likely to lead to increased resistance from these regions.

Control variables related to the national context:

- State capacity:
  - Higher state capacity is expected to be associated with higher compensation, since governments with higher capacity may be more likely to mobilise and distribute higher amounts of funding.
- Economic capacity:
  - Higher GDP is expected to be associated with higher compensation, because larger economies are more likely to mobilize larger funds for compensation.
  - Higher GDP per capita (PPP) is expected to be associated with higher compensation because it indicates the wealth of a country and wellbeing of its citizens. However, GDP per capita also correlates with the level of democracy, which is expected to decrease the amount of compensation paid. GDP per capita may thus conflate two conflicting mechanisms.
- Access to international funding:
  - Recipient-status of EU/ODA-funding is expected to be associated with higher compensation because international funding may provide additional funds to domestic government capacity, so that governments with otherwise smaller economies or lower wealth receive funding for additional compensation.

There are additional variables that can indicate mechanisms operating within complex socio-political systems and can affect the amount of compensation governments pay for coal phase-out. However, many of these variables are not suitable for systematic comparison across countries. For example, public opposition to coal may be indicative of political motivation to phase-out coal countering the resistance of the coal industry, however, there is no comparable systematic data on such opposition.

Additionally, the distinction of countries by “Varieties of Capitalism” may capture relationships between government and industry, but has not been applied to countries on a global scale. More recently, several studies have started to expand the VoC framework to other contexts, and at the same have expanded the types of VoC categorisations. For example, ref.[103] apply the VoC framework to 61 countries, which they categorise into 9 different classes, including CMEs and LMEs. They cover 29 out of the 43 countries with coal phase-out pledges we identify in our study.

While this means that we cannot include VoC in our multiple variable regression analysis, we conduct a qualitative analysis where we compare countries with coal phase-out pledges and with or without compensation policies with VoC classifications from ref[103] to see whether certain types of VoC are more, or less, likely to have coal phase-out pledges or compensation

policies. We find that countries with coal phase-out pledges fall under six of the nine groups. The groups “Coordinated Market Economies” and “European peripheral economies” include purely EU and EEA countries, of which almost all have coal phase-out pledges (excluding Switzerland and Norway which have no active coal power plants). Among the six countries that are classified as liberal market economies, two do not have a coal phase-out pledges at all (Australia and the US), two have a coal phase-out pledge but no compensation (the UK and New Zealand), and two have coal phase-out pledges and compensation (Canada and Ireland). The five countries with the highest compensation cost, South Korea, Germany, Poland, Vietnam and Indonesia, are classified as four different types of countries: Germany as a coordinated market economy, Poland as a European peripheral economy, Korea as an advanced emerging economy and Indonesia and Vietnam as Emerging Economies.

Based on this analysis, we conclude that the classification of countries by VoC does not seem to significantly affect approaches to coal phase-out in our current sample. However, previous literature suggests that the type of government may affect coal-related policies[99,104]. Further research may thus be required to solidify VoC (or similar) classifications on a global level, and to study the effects of government typologies on coal phase-out negotiations and outcomes in more depth.

Regression analysis and results

We run five iterations of multivariable regression analyses: one with the central compensation estimate as the outcome variable and the central estimate for existing pledge ambition; two where we keep the outcome variable stable and vary avoided emissions estimates using pessimistic and optimistic coal phase-out pledge ambitions respectively; and two where we keep avoided emissions stable and vary our outcome variable using lower and higher compensation estimates respectively. This allows us to test the robustness of our results against these parametric uncertainties (Supplementary Note 2).

We apply the same method to each of the five iterations of our regression analysis: Because we have a relatively small number of cases (39 countries), we test models with a maximum of four independent variables. We only include one variable per mechanism in each model, and exclude all models that do not test for a variable capturing the ambition of coal phase-out since the aim of our regression analysis is to test the effect of the ambition of the coal phase-out pledge compared to other relevant mechanisms. For each iteration, we also test collinearity between the variables and exclude any combination of variables with a collinearity larger than 0.7. For example, we exclude the following combinations of variables for our regression anlaysis with central pledges and central compensation estimates (see also Supplementary Figure 6) (in addition of our other criteria of only including one variable per category in each model):

- Size of the coal power plant fleet and Avoided emissions
- Size of the coal power plant fleet and Coal power generation
- Size of the coal power plant fleet and number of coal jobs
- Amount of domestic coal mining and number of coal jobs
- Coal power generation and Avoided emissions
- Government effectiveness and Hanson and Sigman’s Index

Supplementary Figure 6. Regression correlation plot.

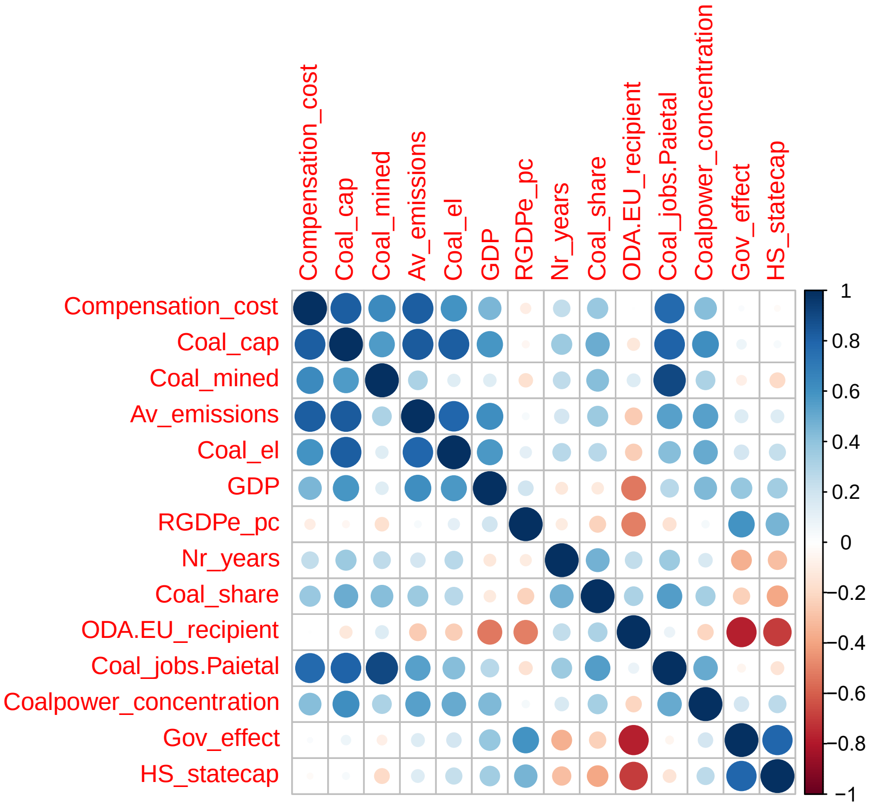

The five regression iterations return 820 machine-generated models in total. In these models, we find that without controlling for access to international funding, there are conflicting results where for example less wealthy countries pay more compensation. We thus pool measures of economic or state capacity with access to international funding, meaning that we include only models with economic or state capacity that also control for access to international funding. We also find conflicting results for the variable share of coal in power generation. Its co-efficients are negative, even though we expect a positive effect on the amount of compensation. This does not reflect any of the presumed mechanisms. One possibility is that the effect of the share of coal power is not as strong as the effect of the size of the power plant fleet, which is captured for example by the avoided emissions variable. We thus exclude models that incorporate these variables.

This results in 330 remaining models; 66 per iteration.

We rank the 66 models from each respective iteration by model fit, measured by the Aikaike Information Criterion (AIC) (Supplementary Tables 12-16).

In all of the five iterations, **avoided emissions** is found significant at the 0.1% level in our ten best-performing models ranked by model fit. The other variable which we test to measure the ambition of the coal phase-out pledge –the number of years over which coal is phased-out – does not occur in any of our ten best-performing models. The highest ranking model this variable appears in is the 15th model (ranked by AIC) in the regression based on pessimistic avoided emissions, and not found to be significant.

Almost all of the top ten models control for a variable related to the strength of the coal sector, most commonly through either the amount of coal mining or the number of coal jobs which are often significant at <0.1% level. The notable exception is the regression analysis with optimistic avoided emissions, where three models do not control for the strength of the coal sector, and in those that do, the amount of coal mined is only significant at the <1% level (Supplementary Tables 12-1). Additionally, the majority of the top ten model in all five iterations control for whether countries are **recipients of ODA or EU funding**.

Based on this analysis, we conclude that avoided emissions, controlled by either the amount of domestic coal mining or the number of coal jobs, as well as whether countries are likely to receive international finance, best predicts the amount of coal phase-out compensation that is paid. Our five regression iterations show that these findings remain robust to uncertainties in the level of existing coal phase-out pledge ambition, and in the level of compensation. Additionally, the finding that avoided emissions is the most significant predictor of the amount of coal phase-out compensation has also stayed robust as the number of our cases has changed throughout writing this article, as new coal phase-out pledges and new compensation policies were announced.

While we based the selection of variables on plausible causal mechanisms that may explain coal phase-out compensation, our regression analysis identifies correlation rather than causation. In-depth case studies of the countries studied may help to validate the results of such a regression analysis.

Supplementary Table 12. Coefficients from ten best-performing regression models with central pledges and central compensation estimates.

"***" indicates that variables are found significant at the 0.1% level. "**" Indicates that variables are found significant at the 1% level. "*" Indicates that variables are found significant at the 5% level. "." Indicates that variables are found significant at above 5% levels.

Variable	*M1*	M2	M3	M4	M5	M6	M7	M8	M9	M10
Avoided emissions (Gt CO2)	*39.8****<br>*(3.8)*	36.7***<br>(4.6)	42.2***<br>(4.2)	34.9***<br>(4.4)	40.3***<br>(3.8)	39.8***<br>(3.9)	40.6***<br>(4.3)	39***<br>(4.6)	37.5***<br>(3.8)	34***<br>(4.5)
Coal mined (Mt)	*0.003****<br>*(0.0001)*		0.003***<br>(0.0005)		0.003***<br>(0.0005)	0.003***<br>(0.0005)	0.003***<br>(0.0005)	0.003***<br>(0.0005)	0.003***<br>(0.0005)	
Coal jobs		0.2***<br>(0.03)		0.2***<br>(0.03)						0.1***<br>(0.03)
Coal power concentration		-9204.<br>(4658)	-5748<br>(4498)	-10460*<br>(4633)			-6908<br>(4583)			
ODA.EU_ recipient	*4044.*<br>*(2065)*	2944<br>(2102)	3659.<br>(2069)		7017*<br>(3238)	5318.<br>(2777)		4432.<br>(2385)		3746.<br>(2146)
GDP								0.0005<br>(0.002)		
Gov_effect					2154<br>(1815)					
SH_cap						1373<br>(1982)				
AIC	*794.4*	794.4	794.6	794.6	794.8	795.8	796	796.3	796.4	796.6
Adj R2	*0.84*	0.85	0.85	0.84	0.85	0.84	0.84	0.84	0.83	0.83

Supplementary Table 13. Coefficients from ten best-performing regression models with pessimistic pledges and central compensation. "***" indicates that variables are found significant at the 0.1% level. "**" Indicates that variables are found significant at the 1% level. "*" Indicates that variables are found significant at the 5% level. "." Indicates that variables are found significant at above 5% levels.

Variable	M1	M2	M3	M4	M5	M6	M7	*M8*	M9	M10
Avoided emissions (Gt CO2)	31.2*** (6.3)	27.3*** (5.9)	30.7*** (6.4)	33.6*** (6.7)	27.6*** (7.3)	31.5*** (6.5)	30.6*** (6.5)	*41*** (6)*	37.3*** (5.7)	37.1*** (7.2)
Coal mined (Mt)								*0.004*** (0.0007)*	0.004*** (0.0007)	0.004*** (0.0007)
Coal jobs	0.2*** (0.03)	0.2*** (0.03)	0.2*** (0.03)	0.2*** (0.03)	0.2*** (0.03)	0.2*** (0.03)	0.2*** (0.03)			
Coal power concentration	-9062 (6019)			-8050 (6082)						
ODA.EU_ recipient			3528 (2770)	2955 (2774)	4738 (3101)	6314 (4342)	4329 (3655)	*4505 (2819)*		5852. (3138)
GDP					0.002 (0.002)					0.002 (0.002)
Gov_effect						1984 (2375)				
SH_cap							0.09 (0.003)			
AIC	813.7	814.1	814.4	814.4	815.5	815.6	816.2	*816.7*	817.4	817.6
Adj R2	0.74	0.73	0.74	0.74	0.74	0.74	0.73	*0.72*	0.71	0.72

Supplementary Table 14. Coefficients from ten best-performing regression models with optimistic pledges and central compensation.

"***" indicates that variables are found significant at the 0.1% level. "**" Indicates that variables are found significant at the 1% level. "*" Indicates that variables are found significant at the 5% level. "." Indicates that variables are found significant at above 5% levels.

Variable	M1	M2	M3	M4	M5	M6	M7	M8	M9	M10
Avoided emissions (Gt CO2)	34.6*** (2.4)	34.9*** (2.4)	33.4*** (2.2)	33.5*** (2.2)	33.3*** (2.2)	32.6*** (2.2)	32.2*** (2.6)	38.5*** (2.3)	36.9*** (2)	39.1*** (2.4)
Coal mined (Mt)	0.001** (0.0004)	0.001** (0.0004)	0.001** (0.0004)	0.001** (0.0004)	0.001** (0.0004)	0.001** (0.0004)	0.001** (0.0004)			
Coal jobs										
Coal power concentration	-6091. (3171)	-5356 (3227)						-4698 (3511)		-5769 (3621)
ODA.EU_ recipient		1657 (1484)	4283* (1979)	4615. (2337)	2160 (1488)		2928 (1761)	5807* (2462)	6031* (2484)	5106* (2141)
GDP							0.0009 (0.001)			
Gov_effect				1804 (1334)				2256 (1435)	2133 (1448)	
SH_cap			2264 (1428)							2504 (1599)
AIC	769.4	770	770.3	771	771	771.3	772.3	776.8	776.8	776.8
Adj R2	0.92	0.92	0.92	0.92	0.91	0.91	0.91	0.9	0.9	0.9

Supplementary Table 15. Coefficients from ten best-performing regression models with central pledges and lower compensation estimates.

"***" indicates that variables are found significant at the 0.1% level. "**" Indicates that variables are found significant at the 1% level. "*" Indicates that variables are found significant at the 5% level. "." Indicates that variables are found significant at above 5% levels.

Variable	M1	M2	M3	M4	M5	M6	M7	M8	M9	M10
Avoided emissions (Gt CO2)	40*** (3.4)	38.3*** (3.1)	37.8*** (3.1)	37.9*** (3.7)	37.8*** (3.1)	36.3*** (3.6)	35.7*** (3.7)	38..7*** (3.4)	36.6*** (3.6)	36*** (3.6)
Coal mined (Mt)	0.001* (0.0004)	0.001* (0.0004)	0.001* (0.0004)		0.001* (0.0004)		0.001* (0.0004)	0.001** (0.0004)		
Coal jobs				0.06* (0.02)		0.07** (0.02)			0.04* (0.02)	0.04* (0.02)
Coal power concentration	-5108 (3622)			-6391. (3774)		-7468. (3765)		-5985 (3672)		
ODA.EU_ recipient	2765 (1666)	5837* (2605)	3108. (1671)	2516 (1703)	4978* (2211)		4054* (1904)		5834* (2666)	0.003. (0.002)
GDP							0.001 (0.001)			
Gov_effect		1978 (1460)							1992 (1485)	
SH_cap					2015 (1578)					
AIC	777.7	777.8	777.9	778	778.1	778.4	778.7	778.7	779.1	779.1
Adj R2	0.85	0.85	0.84	0.85	0.85	0.84	0.84	0.84	0.84	0.84

Supplementary Table 16. Coefficients from ten best-performing regression models with central pledges and higher compensation estimates.

"***" indicates that variables are found significant at the 0.1% level. "**" Indicates that variables are found significant at the 1% level. "*" Indicates that variables are found significant at the 5% level. "." Indicates that variables are found significant at above 5% levels.

Variable	M1	M2	*M3*	M4	M5	M6	M7	M8	M9	M10
Avoided emissions (Gt CO2)	33.6***<br>(6)	35.8***<br>(6.2)	*42.4****<br>*(5.3)*	32.3***<br>(6.1)	45***<br>(5.9)	42.3***<br>(5.3)	28.9***<br>(5.9)	39.4***<br>(5.2)	42.4***<br>(5.4)	33***<br>(6.2)
Coal mined (Mt)			*0.004****<br>*(0.0007)*		0.005***<br>(0.007)	0.005***<br>(0.0007)		0.005***<br>(0.0007)	0.004***<br>(0.0007)	
Coal jobs	0.3***<br>(0.03)	0.2***<br>(0.04)		0.2***<br>(0.04)			0.2***<br>(0.03)			0.2***<br>(0.04)
Coal power concentration	-1344*<br>(6283)	-1192.<br>(6355)			-6160<br>(6274)					
ODA.EU_ recipient		3555<br>(2867)	*5214.*<br>*(2853)*	4594<br>(2913)	4801<br>(2885)	8488.<br>(4508)			5838<br>(3860)	7772.<br>(4594)
Gov_effect						2372<br>(2527)				2294<br>(2559)
SH_cap									672<br>(2755)	
AIC	818.4	818.6	*819.6*	820.5	820.5	820.6	821.1	821.2	821.5	821.6
Adj R2	0.82	0.82	*0.81*	0.81	0.81	0.81	0.8	0.8	0.8	0.8

Supplementary Note 4. Just transitions for coal phase-out

The concept of a "just transition" originated in labor unions in the US and Canada in the 1980s, who demanded support as their industries were downsized[105–108] and workers faced the risk of unemployment. International labor unions later adopted the idea including in international climate negotiations where they promoted the concept in connection to low-carbon transitions and their effects on carbon-intensive industries[107,109]. Originally, this approach highlighted the importance of helping workers to find new jobs through re-training or supporting the local economy to restore jobs[108,109]. More recently, the concept of just transitions has been expanded beyond its original notion of employment effects by energy and climate scholars to include the broader societal distribution of the costs and benefits of low-carbon transitions beyond effects on workers, including on future generations, the potential of low-carbon transitions to alleviate existing societal inequalities (such as gender inequality or energy poverty), and the effect of low-carbon transitions on inequalities between the Global North and the Global South [107,108,110]. Through this expansion, a number of different dimensions of justice have been identified and debates have emerged about which actors should be included in just transitions processes and benefit from them, and how just transitions relate to the speed of transitions[107,110–112] .

In this note, we first show interpretations of the just transition concept across different types of organisations. We then synthesize the different justice dimensions and relate these dimensions to coal phase-out. Then, we discuss coal phase-out compensation and how our research informs the relationship between coal phase-out compensation and different dimensions of justice. We conclude with raising additional questions for further research on this topic.

Supplementary Table 17 shows that while there are some commonalities among different interpretations of just transition (such as a link between societal justice and environmental protection, and a link to the SDGs), there are also differences. Some organisations focus on establishing equality and justice globally, and on national level (specifically the CDP)[113], while others highlight specific subnational actors such as workers (especially the ILO)[114]. Some also consider companies and industries as actors that need to be considered within just transitions (for example the EBRD and the ILO)[114,115], while others highlight that such actors may need to pay themselves (such as the WWF)[116].

Supplementary Table 17. Principles and definitions of just transition in different organisations.

Organisation name	Main principles and/or definition of just transition
International Labor Organization (ILO)	Main principles of just transition[114]:<br>- social dialogue with all relevant stakeholders<br>- fundamental principles and rights at work and promoting the creation of decent jobs,<br>- recognizing the gender dimension of environmental challenges,<br>- providing "an enabling environment for enterprises, investors and consumers to embrace and drive the transition", considering country-specific conditions, including their stage of development, and<br>- fostering international cooperation among countries.
United Nation's Committee for Development Policy (CDP)	Definition: "A just transition [ensures] no one is left [or] pushed behind in the transition[,] can enable more ambitious climate action and provide an impetus to attaining the Sustainable Development Goals."[113]<br><br>Main principles:<br>- inclusive dialogue,<br>- reflection of countries' current needs as well as historical responsibilities for climate change with open policy space for developing countries to develop productive capacities;<br>- consideration of global climate justice and common but differentiated responsibilities;<br>- countries should meet climate commitments without creating barriers to trade for poorer countries or excluding them from opportunities associated with the expansion of new product markets;<br>- provision of new intellectual property frameworks for and the co-development of clean technologies, the expansion of systems for the payment of ecosystem services and scaled-up international cooperation to ensure financing for infrastructure and resilience-building.
European Bank for Reconstruction and Development (EBRD)	Definition: "A just transition seeks to ensure that the substantial benefits of a green economy transition are shared widely, while also supporting those who stand to lose economically – be they countries, regions, industries, communities, workers or consumers. […]The just transition concept links to 14 of the 17 Sustainable Development Goals, explicitly drawing together SDGs 12 – climate action, 10 – reduced inequalities, 8 – decent work and economic growth, and 7 - affordable and clean energy."[115]
World Wide Fund for Nature (WWF)	Principles that territorial just transition plans are assessed against[116]:<br>- Climate ambition,<br>- Fossil fuel phase-out,<br>- Sustainable economic diversification,<br>- Addressing inequalities,<br>- Supporting environmental objectives,<br>- Polluter pays,<br>- Adequate public and private funding,<br>- Participatory processes,<br>- Place-based approach,<br>- Review and indicators

The contestedness of the just transition concept is also recognized in the literature[107]. Scholars frequently highlight several dimensions of justice: procedural, recognitional, distributional, and restorative[108,117–119]. Procedural justice broadly relates to the participation of different groups in negotiation, decision-making and implementation processes aimed at ensuring a just transition[108,117]. Recognition-based justice is often included as a sub-component of procedural justice, relating to whether all relevant groups have been recognized within a participatory process[120]. Restorative justice relates to addressing losses due to social and economic changes such as energy transitions; this can relate to job losses but also environmental damages from the fossil fuel-based technology[108]. Finally, distributional justice covers the distribution of costs and benefits of both climate change impacts and climate change mitigation across different groups[108,119].

All justice dimensions are relevant to coal phase-out. In terms of procedural justice, there are debates about which actors should be allowed to participate in just transition processes. For example, ref. [111] argue that fossil fuel companies are adversely affected by low-carbon transitions and thus should be able to participate in just transition processes. The inclusion of such actors in just transition processes can however also lead to further justice concerns, if "power imbalances [remain unchecked and] decisionmaking power [is shifted] towards incumbents"[121].

This relates to the recognitional dimension of justice which is to what extent specific damages and inequalities are recognized. Different interpretations of just transitions recognize different damages and inequalities: for example, while some organisations, scholars and just transition strategies consider industries under "those who stand to lose economically"[115] and include them under those to be supported under just transitions[111,115,122], others measure just transitions against whether the polluting industry itself is made to pay[116].

The recognition of specific injustices also influences the restorative justive dimension and whether just transition is interpreted as restoring jobs to previous levels in line with traditional interpretations of the just transition concept[123], restoring damages or inequalities that resulted from coal production and use itself such as rehabilitating former mining areas[107–109], or restoring broader societal injustices and inequalities beyond such as gender inequality[121]. Finally, the distributional justice dimension in relation to coal phase-out can be considered on different geographical scales. The distribution of costs and benefits of coal phase-out can relate to certain countries or regions bearing the brunt of losses in jobs and tax revenues, to companies needing to close, or to consumers facing higher electricity cost[111]. The types of costs may also differ across contexts - for some countries in the Global South, coal phase-out is likely to relate to the challenge of providing universal access to clean energy and electricity, which is not a major concern in the Global North.

Financial compensation may help to re-distribute costs and benefits of coal phase-outs and thus address restorative and distributional issues which arise from coal phase-out. However, the processes for developing such policies, and the outcomes of these processes, can also be questioned under the procedural and recognitional justice dimensions arising from which actors have been involved in negotiations around coal phase-out compensation, and whether power imbalances may have influenced the negotiations[111,121]. By quantifying the outcomes of compensation processes, we lay the foundation to understanding whether, and how, different procedures lead to different outcomes. Future research may map whether different

approaches to formulating just transition strategies (such as the forming of commissions, compared to parliamentary or bilateral negotiations for coal phase-out compensation) lead to different outcomes (such as different beneficiaries, or different amounts of compensation).

The amount of compensation paid to different actors may also be assessed under the distributional and restorative justice dimensions. Understanding how much compensation each group receives helps to understand how (and whether) compensation policies re-*distribute* the costs and benefits of coal phase-out across different groups. Figure 2 shows that most flows support economic and environmental recovery in coal regoins, followed by power plant and mining companies, and support of renewables and low-carbon infrastructure. Workers receive the least amount of direct compensation, even though some regional funding may also provide new jobs and retraining opportunities which can ultimately benefit workers. Additionally, Figure 2 shows that a large amount of funding is also paid to national governments of major coal consumers in the Global South. Our work thus shows that the outcomes of political negotiations around just transition policies have coalesced to supporting regions and countries dependent on coal power.

Future research may further investigate compensation policies from a restorative perspective. Currently, it is unclear to what extent compensation policies are able to restore damages related to coal phase-out – will they lead to sustained and sufficient gains in job opportunities, for example? Such research will be possible as just transition policies shift from the planning to the implementation stage. With the expansion of just energy transition partnerships to countries of the Global South (see Supplementary Note 1), ongoing discussions of just transition in India[124], and calls to implement just transition strategies in China[125–128], questions also arise around how the justice challenges from coal phase-out in these regions compare to justice challenges in the Global North. Ref.[128] for example propose that compensatory policies might be important in the Chinese context specifically targeting coal-dependent regions. Wong et al[129] estimate that lessons from rural contexts in Europe may be applicable to regions such as Inner Mongolia and Shandong in China. However, future research may compare existing justice challenges and inequalities between contexts to better understand what lessons from just transition processes and outcomes are transferrable to which contexts.

Supplementary References

1. Vinichenko, V., Vetier, M., Jewell, J., Nacke, L. & Cherp, A. Phasing out coal for 2 °C target requires worldwide replication of most ambitious national plans despite security and fairness concerns. *Environ Res Lett* **18**, 014031 (2023).

2. OECD. OECD Inventory of Support Measures for Fossil Fuels. https://www.oecd.org/fossil-fuels/countrydata/ (2021).

3. Gençsü, I. & Roberts, L. *G20 Coal Subsidies: Tracking Government Support to a Fading Industry | ODI: Think Change*. https://odi.org/en/publications/g20-coal-subsidies-tracking-government-support-to-a-fading-industry/ (2019).

4. IMF. World Economic Outlook Database, October 2022. https://www.imf.org/en/Publications/WEO/weo-database/2022/October (2022).

5. OECD. Aid (ODA) disbursements to countries and regions [DAC2a]. https://stats.oecd.org/Index.aspx?DataSetCode=TABLE2A (2023).

6. Rathi, A. & Chaurdhary, A. India Wants $1 Trillion Before It Raises Targets to Cut Emissions. *Bloomberg* https://www.bloomberg.com/news/articles/2021-11-10/india-holds-back-on-climate-pledge-until-rich-nations-pay-1-trillion (2021).

7. OECD. *Climate Finance Provided and Mobilised by Developed Countries in 2016-2020*. 76 https://doi.org/10.1787/286dae5d-en (2022).

8. Council of Ministers of the Republic of Bulgaria. National Recovery and Resilience Plan of the Republic of Bulgaria. https://nextgeneration.bg/14 (2021).

9. The European Parliament & The Council. Regulation of the European Parliament and of the Council establishing the Just Transition Fund. https://data.consilium.europa.eu/doc/document/PE-5-2021-INIT/en/pdf (2021).

10. European Commission. *European Semester 2020 Overview of Investment Guidance on the Just Transition Fund 2021-2027 per Member State (Annex D)*. https://commission.europa.eu/system/files/2020-02/annex_d_crs_2020_en.pdf (2020).

11. Kojouharova, V. *What Is the Current State of the Just Transition Processes in Bulgaria? - Bankwatch*. https://bankwatch.org/publication/what-is-the-current-state-of-the-just-transition-processes-in-bulgaria (2023).

12. Government of Canada. *People-Centred Just Transition: Discussion Paper*. https://publications.gc.ca/collections/collection_2021/rncan-nrcan/M4-210-2021-eng.pdf (2021).

13. Canada, D. of F. Investing in the Middle Class: Budget 2019. https://www.budget.canada.ca/2019/home-accueil-en.html (2019).

14. Audit and Evaluation Branch Natural Resources Canada. Evaluation of the Regional Electricity Cooperation and Strategic Infrastructure (RECSI) Initiative. *Government of Canada* https://www.nrcan.gc.ca/transparency/reporting-and-accountability/plans-and-performance-reports/strategic-evaluation-division/reports-and-plans-year/evaluation-the-regional-electricity-cooperation-and-strategic (2019).

15. Task Force on Just Transition for Canadian Coal Power Workers and Communities. *A Just and Fair Transition for Canadian Coal Power Workers and Communities*. https://publications.gc.ca/collections/collection_2019/eccc/En4-361-2019-eng.pdf (2018).

16. Newfoundland Labrador *et al. Pan-Canadian Framework on Clean Growth and Climate Change: Canada's Plan to Address Climate Change and Grow the Economy*. https://publications.gc.ca/collections/collection_2017/eccc/En4-294-2016-eng.pdf (2016).

17. European Commission. Investing in skills and jobs for the green transition in Croatia | European Social Fund Plus. https://ec.europa.eu/european-social-fund-plus/en/news/skills-and-jobs-green-transition-croatia (2022).

18. European Commission. 2021-2027 JTF Finances Details | Data | European Structural and Investment Funds. https://cohesiondata.ec.europa.eu/2021-2027-Categorisation/2021-2027-JTF-Finances-Details/dy7j-i7p6 (2023).

19. Ministry of Industry and Trade. Dokumenty | Národní plán obnovy. https://www.planobnovycr.cz/dokumenty (2021).

20. European Commission. EU Cohesion Policy: €1.64 billion for Czechia. https://ec.europa.eu/commission/presscorner/detail/en/IP_22_5702 (2022).

21. Ministry of the Environment of the Czech Republic. Operational programme Just Transition (OPJT) 2021-2027. https://www.mzp.cz/en/operational_programme_just_transition (2023).

22. Ministry of Economic Affairs and Employment. Investment incentives to promote rapid phase-out of coal in energy production. https://valtioneuvosto.fi/en/-/1410877/investment-incentives-to-promote-rapid-phase-out-of-coal-in-energy-production (2020).

23. Ministry of Economic Affairs and Employment. Draft Budget of Ministry of Economic Affairs and Employment for 2021: building new growth pathways. https://valtioneuvosto.fi/en/-/1410877/draft-budget-of-ministry-of-economic-affairs-and-employment-for-2021-building-new-growth-pathways (2020).

24. Ministère de l'économie des finances et de la souveraineté industrielle et numérique. Budget Général Mission ministérielle Projects annuels de performances Annexe au projet de loi de finances pour 2021: Programme 174 Énergie, climat et après-mines. https://www.budget.gouv.fr/documentation/documents-budgetaires/exercice-2021/projet-de-loi-de-finances/budget-general/ecologie-developpement-et-mobilite-durables (2021).

25. Ministère de la transition écologique et solidaire. *Fermeture Des Centrales a Charbon d'ici 2022 - Enjeux et Projets de Territoire*. https://www.ecologie.gouv.fr/sites/default/files/DP_Fermeture%20des%20centrales%20a%2

0charbon%20d%27ici%202022%20-%20Enjeux%20et%20projets%20de%20territoire.pdf (2020).

26. Bundestag. *Gesetz Zur Reduzierung Und Zur Beendigung Der Kohleverstromung Und Zur Anderung Weiterer Gesetze*. (2020).

27. Tiedemann, S. & Müller-Hansen, F. Auctions to phase out coal power: Lessons learned from Germany. *Energ Policy* **174**, 113387 (2023).

28. Bundesregierung. Investitionsgesetz Kohleregionen vom 8. August 2020 (BGBl. I S. 1795). https://www.gesetze-im-internet.de/invkg/InvKG.pdf (2020).

29. European Commission. EU Cohesion Policy: €2.5 billion for Germany. https://ec.europa.eu/commission/presscorner/detail/en/ip_22_6275 (2022).

30. ZEIT Online. Gesetz zum Kohleausstieg: Kabinett beschließt Kohleausstieg bis 2038 | ZEIT ONLINE. *DIE ZEIT* (2020).

31. Ministry of Environment and Energy. *Just Transition Development Plan of Lignite Areas*. https://www.sdam.gr/sites/default/files/consultation/Master_Plan_Public_Consultation_ENG.pdf (2020).

32. European Commission. EU cohesion policy: €1.63 billion for TJTP in Greece. https://ec.europa.eu/commission/presscorner/detail/en/ip_22_3711 (2022).

33. OECD. *OECD Environmental Performance Reviews: Greece 2020*. (2020).

34. European Commission. EU Cohesion Policy 2021-2027: Hungary. https://ec.europa.eu/commission/presscorner/detail/en/ip_22_7801 (2022).

35. European Commission. Just Energy Transition Partnership with Indonesia. https://ec.europa.eu/commission/presscorner/detail/en/IP_22_6926 (2022).

36. European Commission. EU Cohesion Policy: €1 billion for Italy. https://ec.europa.eu/commission/presscorner/detail/en/ip_22_7800 (2022).

37. European Commission. State Aid SA.54537 (2020/NN) - Netherlands Prohibition of coal for the production of electricity in the Netherlands. https://ec.europa.eu/competition/state_aid/cases1/202025/284556_2165085_151_2.pdf (2020).

38. S&P Global Commodity Insights. Dutch government agrees on closure compensation for 731-MW Rotterdam coal plant. *S&P Global Commodity Insights* https://www.spglobal.com/commodityinsights/en/market-insights/latest-news/electric-power/120121-dutch-government-agrees-on-closure-compensation-for-731-mw-rotterdam-coal-plant (2021).

39. Meijer, B. & Potter, M. Dutch court denies RWE and Uniper compensation for closure of coal plants | Reuters. *Reuters* (2022).

40. Czyżak, P. *et al. Poland's Planned Coal Monopoly – Who Pays the Price?* https://instrat.pl/wp-content/uploads/2020/12/CE_Instrat_Coal-Monopoly_3.12.2020.pdf (2020).

41. European Commission. State Aid SA.41161 (2015/N) – Poland State aid to Polish coal mining in the period 2015-2018. https://ec.europa.eu/competition/state_aid/cases/257337/257337_1866791_407_2.pdf (2016).

42. European Commission. €3.85 billion for a just transition in five Polish regions. https://ec.europa.eu/commission/presscorner/detail/en/ip_22_7413 (2022).

43. European Commission. €223.8 million for a just climate transition in Portugal. https://ec.europa.eu/commission/presscorner/detail/en/ip_22_7718 (2022).

44. European Commission. State Aid SA.55038 (2019/N) – Slovakia – Aid to cover the exceptional costs of Hornonitrianske bane Prievidza (HBP) related to the closure of its mining operations. https://ec.europa.eu/competition/state_aid/cases1/20205/281626_2128088_142_2.pdf (2019).

45. European Commission. €459 million for a just climate transition in Slovakia. https://ec.europa.eu/commission/presscorner/detail/en/ip_22_7086 (2022).

46. European Commission. Cohesion Policy: More than €258 million for Slovenia. https://ec.europa.eu/commission/presscorner/detail/en/ip_22_7744 (2022).

47. Council of Ministers. Government of Spain is taking the necessary measures to protect the integrity of its borders [Government/Activity of the Council of Ministers]. https://www.lamoncloa.gob.es/lang/en/gobierno/councilministers/paginas/2021/20210518council.aspx (2021).

48. El Instituto para la Reestructuración de la Minería del Carbón y Desarrollo Alternativo de las Comarcas Mineras. *Just Transition Agreements Update September 2021*. https://www.transicionjusta.gob.es/Documents/Convenios_transicion_justa/common/Folleto_Convenios_Transicion_Justa_EN_uv.pdf (2021).

49. European Commission. EU Cohesion policy: €869 million for Spain. https://ec.europa.eu/commission/presscorner/detail/en/ip_22_7868 (2022).

50. European Commission. Commission Staff Working Document Analysis of the recovery and resilience plan of Romania. https://eur-lex.europa.eu/legal-content/EN/TXT/PDF/?uri=CELEX:52021SC0276&from=EN (2021).

51. European Commission. Inforegio - EU Cohesion Policy: €2.14 billion for a just climate transition in Romania. https://ec.europa.eu/regional_policy/whats-new/newsroom/12-09-2022-eu-cohesion-policy-eur2-14-billion-for-a-just-climate-transition-in-romania_en (2022).

52. Government of the Republic of Korea. Korean New Deal. https://english.moef.go.kr/pc/selectTbPressCenterDtl.do?boardCd=N0001&seq=4948 (2020).

53. Foreign & Commonwealth and Development Office. Political declaration on establishing the Just Energy Transition Partnership with Viet Nam - GOV.UK. https://www.gov.uk/government/publications/vietnams-just-energy-transition-partnership-political-declaration/political-declaration-on-establishing-the-just-energy-transition-partnership-with-viet-nam (2022).

54. OECD. DAC List of ODA Recipients. https://www.oecd.org/dac/financing-sustainable-development/development-finance-standards/daclist.htm (2023).

55. Busch, B., Kauder, B. & Sultan, S. *Wer Finanziert Die EU? Nettozahler Und Nettoempfänger Der EU.* https://www.iwkoeln.de/fileadmin/user_upload/Studien/Report/PDF/2022/IW-Report_2022-Wer-finanziert-die-EU.pdf (2022).

56. S&P Global. *World Electric Power Plants Database*. (2021).

57. International Monetary Fund & International Financial Statistics. Official exchange rate (LCU per US$, period average) | Data. https://data.worldbank.org/indicator/PA.NUS.FCRF (2022).

58. Novinite. American AES Launches Bulgaria’s Galabovo TPP - Novinite.com - Sofia News Agency. https://www.novinite.com/articles/128908/American+AES+Launches+Bulgaria’s+Galabovo+TPP (2011).

59. Mizzy, S. The launch of the modernized Prunéřov is hampered by the insolvency of the main suppliers – europe-cities.com. https://europe-cities.com/2016/11/13/the-launch-of-the-modernized-prunerov-is-hampered-by-the-insolvency-of-the-main-suppliers/ (2016).

60. Power Technology. Ledvice Power Station Expansion, Czech Republic. https://www.power-technology.com/marketdata/ledvice-power-station-expansion-czech-republic/ (2022).

61. Vattenfall. Vattenfall’s investments 2000 – 2016. From fossil fuel to wind - The history and heritage of Vattenfall. https://history.vattenfall.com/stories/from-hydro-power-to-solar-cells/vattenfalls-investments-2000-2016-from-fossil-fuel-to-wind.

62. Enerdata. Germany rules that approval for Datteln-4 coal-fired plant was invalid | Enerdata. https://www.enerdata.net/publications/daily-energy-news/germany-rules-approval-datteln-4-coal-fired-plant-was-invalid.html (2021).

63. Power Technology. GKM Power Plant Unit 9, Germany. https://www.power-technology.com/marketdata/gkm-power-plant-unit-9-germany/ (2021).

64. Eckert, V. Trianel calls for German power market reform | Reuters. *Reuters* https://www.reuters.com/article/germany-power-trianel-idINL5N0B588P20130205 (2013).

65. Beyond Coal. Vattenfall requests closure of Germany’s second-youngest coal power plant - Europe Beyond Coal : Europe Beyond Coal. https://beyond-coal.eu/2020/09/04/vattenfall-requests-closure-of-germanys-second-youngest-coal-power-plant/ (2020).

66. Power Technology. Neurath F&G (BoA 2&3) Power Plant, Germany. https://www.power-technology.com/marketdata/neurath-fg-boa-23-power-plant-germany/ (2021).

67. Power Technology. Rheinhafen Karlsruhe RDK 8 Power Station, Germany. https://www.power-technology.com/marketdata/rheinhafen-karlsruhe-rdk-8-power-station-germany/ (2021).

68. Die Presse. EVN enthält für Mängel bei "Walsum 10" Entschädigung | DiePresse.com. https://www.diepresse.com/5123426/evn-enthaelt-fuer-maengel-bei-walsum-10-entschaedigung (2016).

69. Andresen, T. & Zha, W. This €1 Billion Power Plant May Never Be Switched on - Bloomberg. https://www.bloomberg.com/news/articles/2015-12-23/brand-new-rwe-plant-is-latest-victim-of-merkel-s-energy-shift#xj4y7vzkg (2015).

70. Power Technology. Wilhelmshaven Coal Fired Power Plant, Germany. https://www.power-technology.com/marketdata/wilhelmshaven-coal-fired-power-plant-germany/ (2021).

71. Wynn, G. The big Dutch coal power mistake and what it means for Europe. *Energy Post* https://energypost.eu/big-dutch-coal-mistake-future-coal-europe-2/ (2016).

72. Darby, M. Dutch coal plant lost €800m in value last year. https://www.climatechangenews.com/2017/04/10/dutch-coal-plant-lost-e800m-value-one-year/ (2017).

73. Modern Power Systems. 858 MWe supercritical extension for Belchatow - Modern Power Systems. https://www.modernpowersystems.com/features/feature858-mwe-supercritical-extension-for-belchatow/ (2007).

74. Power Technology. PKE Jaworzno III Power Plant II, Poland. https://www.power-technology.com/marketdata/pke-jaworzno-iii-power-plant-ii-poland/ (2021).

75. NS Energy. Opole Power Plant- Europe's biggest coal power project completed in 2019. https://www.nsenergybusiness.com/projects/opole-power-plant-expansion/.

76. Power Technology. PGE Turow Power Plant (Expansion), Poland. https://www.power-technology.com/marketdata/pge-turow-power-plant-expansion-poland/ (2022).

77. CEE Bankwatch Network. Sostanj lignite thermal power plant unit 6, Slovenia - Bankwatch. https://bankwatch.org/project/sostanj-lignite-thermal-power-plant-unit-6-slovenia.

78. European Commission. South Africa Just Energy Transition Investment Plan. https://ec.europa.eu/commission/presscorner/detail/en/STATEMENT_22_6664 (2022).

79. Bhushan, C. *Just Transition, Just Finance: Methodology and Costs for Just Energy Transition in India*. https://iforest.global/events/global-report-launch-webinar-just-transition-just-finance/ (2023).

80. Sarr, S. & Fall, S. Just energy transitions and partnerships in Africa: A Senegal case study | IDDRI. https://www.iddri.org/en/publications-and-events/report/just-energy-transitions-and-partnerships-africa-senegal-case-study (2022).

81. Kramer, K. Just Energy Transition Partnerships: An opportunity to leapfrog from coal to clean energy | International Institute for Sustainable Development. https://www.iisd.org/articles/insight/just-energy-transition-partnerships (2022).

82. UK COP 26. Political declaration on the just energy transition in South Africa. https://ukcop26.org/political-declaration-on-the-just-energy-transition-in-south-africa/ (2021).

83. Mathiesen, K. & Barigazzi, J. G7 offered Vietnam and Indonesia $15B to drop coal. They said 'maybe' – POLITICO. *POLITICO* https://www.politico.eu/article/g7-climate-change-energy-crisis-vietnam-and-indonesia-15b-to-drop-coal-they-said-maybe/ (2022).

84. European Commission. Just Energy Transition Partnership with Senegal. https://ec.europa.eu/commission/presscorner/detail/en/ip_23_3448 (2023).

85. Naudé, L. Just Energy Transition Partnership offers should come as grants, not loans | WWF South Africa. *WWF* https://www.wwf.org.za/?41686/Just-Energy-Transition-Partnership-offers-should-come-as-grants-not-loans (2022).

86. Fitch Ratings. Debt-Funded Energy Transition Schemes Unlikely to Weigh on EM Credit Profiles. https://www.fitchratings.com/research/sovereigns/debt-funded-energy-transition-schemes-unlikely-to-weigh-on-em-credit-profiles-11-11-2022 (2022).

87. Presse- und Informationsamt der Bundesregierung. Kohleausstieg und Strukturwandel | Bundesregierung. https://www.bundesregierung.de/breg-de/themen/klimaschutz/kohleausstieg-1664496 (2023).

88. Hatherick, V. Cop 27: Envoy signals Germany is credible in coal talks | Argus Media. *Argus Media* https://www.argusmedia.com/en/news/2389791-cop-27-envoy-signals-germany-is-credible-in-coal-talks (2022).

89. UNFCCC. Global coal to clean power transition statement. https://ukcop26.org/global-coal-to-clean-power-transition-statement/ (2021).

90. Brutschin, E., Schenuit, F., Ruijven, B. van & Riahi, K. Exploring Enablers for an Ambitious Coal Phaseout. *Politics Gov* **10**, 200–212 (2022).

91. Jewell, J., Vinichenko, V., Nacke, L. & Cherp, A. Prospects for powering past coal. *Nat Clim Change* **9**, 592–597 (2019).

92. Blondeel, M., Graaf, T. V. de & Haesebrouck, T. Moving beyond coal: Exploring and explaining the Powering Past Coal Alliance. *Energy Res Soc Sci* **59**, 101304 (2020).

93. Lægreid, O. M., Cherp, A. & Jewell, J. Coal phase-out pledges follow peak coal: evidence from 60 years of growth and decline in coal power capacity worldwide. *Oxf. Open Energy* **2**, oiad009 (2023).

94. IEA. *Coal in Net Zero Transitions: Strategies for Rapid, Secure and People-Centred Change*. https://iea.blob.core.windows.net/assets/08119e54-73ce-4207-b6ce-fbe75c241191/CoalinNetZeroTransitions.pdf (2022).

95. Skjølsvold, T. M. & Coenen, L. Are rapid and inclusive energy and climate transitions oxymorons? Towards principles of responsible acceleration. *Energy Res Soc Sci* **79**, 102164 (2021).

96. Diluiso, F. *et al.* Coal transitions—part 1: a systematic map and review of case study learnings from regional, national, and local coal phase-out experiences. *Environ Res Lett* **16**, 113003 (2021).

97. Erickson, P., Kartha, S., Lazarus, M. & Tempest, K. Assessing carbon lock-in. *Environ Res Lett* **10**, 084023 (2015).

98. Malik, A. *et al.* Reducing stranded assets through early action in the Indian power sector. *Environ Res Lett* **15**, 094091 (2020).

99. Rentier, G., Lelieveldt, H. & Kramer, G. J. Varieties of coal-fired power phase-out across Europe. *Energ Policy* **132**, 620–632 (2019).

100. Brauers, H., Oei, P.-Y. & Walk, P. Comparing coal phase-out pathways: The United Kingdom's and Germany's diverging transitions. *Environ Innovation Soc Transitions* **37**, 238–253 (2020).

101. Brauers, H. & Oei, P.-Y. The political economy of coal in Poland: Drivers and barriers for a shift away from fossil fuels. *Energ Policy* **144**, 111621 (2020).

102. Stognief, N., Walk, P., Schöttker, O. & Oei, P.-Y. Economic Resilience of German Lignite Regions in Transition. *Sustainability-basel* **11**, 5991 (2019).

103. Witt, M. A. *et al.* Mapping the business systems of 61 major economies: a taxonomy and implications for varieties of capitalism and business systems research. *Socio-Econ. Rev.* **16**, 5–38 (2017).

104. Jakob, M., Flachsland, C., Steckel, J. C. & Urpelainen, J. Actors, objectives, context: A framework of the political economy of energy and climate policy applied to India, Indonesia, and Vietnam. *Energy Res Soc Sci* **70**, 101775 (2020).

105. Galgóczi, B. Just transition on the ground: Challenges and opportunities for social dialogue. *Eur J Ind Relat* **26**, 367–382 (2020).

106. Rector, J. The Spirit of Black Lake: Full Employment, Civil Rights, and the Forgotten Early History of Environmental Justice. *Mod Am Hist* **1**, 45–66 (2018).

107. Stevis, D. & Felli, R. Planetary just transition? How inclusive and how just? *Earth Syst Gov* **6**, 100065 (2020).

108. McCauley, D. & Heffron, R. Just transition: Integrating climate, energy and environmental justice. *Energ Policy* **119**, 1–7 (2018).

109. Stevis, D. & Felli, R. Global labour unions and just transition to a green economy. *Int Environ Agreements Politics Law Econ* **15**, 29–43 (2015).

110. Newell, P. & Mulvaney, D. The political economy of the 'just transition.' *Geogr J* **179**, 132–140 (2013).

111. Green, F. & Gambhir, A. Transitional assistance policies for just, equitable and smooth low-carbon transitions: who, what and how? *Clim. Polic.* **20**, 902–921 (2020).

112. Newell, P. J., Geels, F. W. & Sovacool, B. K. Navigating tensions between rapid and just low-carbon transitions. *Environ Res Lett* **17**, 041006 (2022).

113. Committee for Development Policy. *Just Transition*. https://www.un.org/development/desa/dpad/wp-content/uploads/sites/45/CDP-excerpt-2023-1.pdf (2023).

114. International Labour Organization. *Guidelines for a Just Transition towards Environmentally Sustainable Economies and Societies for All*. https://www.ilo.org/wcmsp5/groups/public/@ed_emp/@emp_ent/documents/publication/wcms_432859.pdf (2015).

115. European Bank for Reconstruction and Development. What is a just transition? https://www.ebrd.com/what-we-do/just-transition.

116. World Wide Fund for Nature. *Summary Report Territorial Just Transition Plan Scorecard Assessment*. https://wwfeu.awsassets.panda.org/downloads/wwf___tjtp_v03_final.pdf (2023).

117. Bang, G., Rosendahl, K. E. & Böhringer, C. Balancing cost and justice concerns in the energy transition: comparing coal phase-out policies in Germany and the UK. *Clim. Polic.* **22**, 1000–1015 (2022).

118. Harrahill, K. & Douglas, O. Framework development for 'just transition' in coal producing jurisdictions. *Energ Policy* **134**, 110990 (2019).

119. Gürtler, K., Beer, D. L. & Herberg, J. Scaling just transitions: Legitimation strategies in coal phase-out commissions in Canada and Germany. *Political Geogr.* **88**, 102406 (2021).

120. Sovacool, B. K., Heffron, R. J., McCauley, D. & Goldthau, A. Energy decisions reframed as justice and ethical concerns. *Nat Energy* **1**, 16024 6 (2016).

121. Hauenstein, C., Braunger, I., Krumm, A. & Oei, P.-Y. Overcoming political stalemates: The German stakeholder commission on phasing out coal. *Energy Res. Soc. Sci.* **103**, 103203 (2023).

122. European Commission. European Semester 2020: Overview of Investment Guidance on the Just Transition Fund 2021-2027 per Member State (Annex D). https://commission.europa.eu/system/files/2020-02/annex_d_crs_2020_en.pdf (2020).

123. Heffron, R. J. & McCauley, D. What is the 'Just Transition'? *Geoforum* **88**, 74–77 (2018).

124. iForest (International Forum for Environment, Sustainability and Technology). National report release-Korba: Planning a Just Transition for India's Biggest Coal and Power District - iFOREST - International Forum for Environment, Sustainability & Technology. https://iforest.global/events/national-report-release-korba-planning-a-just-transition-for-indias-biggest-coal-and-power-district/ (2022).

125. He, G. *et al.* Enabling a Rapid and Just Transition away from Coal in China. *One Earth* **3**, 187–194 (2020).

126. Clark, A. & Zhang, W. Estimating the Employment and Fiscal Consequences of Thermal Coal Phase-Out in China. *Energies* **15**, 800 (2022).

127. Zhang, X. *et al.* Immediate actions on coal phaseout enable a just low-carbon transition in China's power sector. *Appl Energ* **308**, 118401 (2022).

128. Voss, B. H. van & Rafaty, R. Sensitive intervention points in China's coal phaseout. *Energ Policy* **163**, 112797 (2022).

129. Wong, J., Röser, F. & Maxwell, V. *Coal Phase-out and Just Transitions Lessons Learned from Europe*. https://newclimate.org/sites/default/files/2022-11/coal_phase_out_paper_nov_2022.pdf (2022).
