# Supplementary material for: Compensating affected parties necessary for rapid coal phase-out but expensive if extended to major emitters
Source: Nat Commun. 2024 May 7;15:3742. doi: 10.1038/s41467-024-47667-w (PMC11076460; doi:10.1038/s41467-024-47667-w)
Supplement: Supplementary file 3 — Reporting Summary [file 41467_2024_47667_MOESM3_ESM.pdf]

nature portfolio

Corresponding author(s): Jessica Jewell

Last updated by author(s): Mar 8, 2024

Reporting Summary

Nature Portfolio wishes to improve the reproducibility of the work that we publish. This form provides structure for consistency and transparency in reporting. For further information on Nature Portfolio policies, see our Editorial Policies and the Editorial Policy Checklist.

Statistics

For all statistical analyses, confirm that the following items are present in the figure legend, table legend, main text, or Methods section.

n/a	Confirmed	
☐	☒	The exact sample size (*n*) for each experimental group/condition, given as a discrete number and unit of measurement
☐	☒	A statement on whether measurements were taken from distinct samples or whether the same sample was measured repeatedly
☐	☒	The statistical test(s) used AND whether they are one- or two-sided<br>*Only common tests should be described solely by name; describe more complex techniques in the Methods section.*
☐	☒	A description of all covariates tested
☐	☒	A description of any assumptions or corrections, such as tests of normality and adjustment for multiple comparisons
☐	☒	A full description of the statistical parameters including central tendency (e.g. means) or other basic estimates (e.g. regression coefficient) AND variation (e.g. standard deviation) or associated estimates of uncertainty (e.g. confidence intervals)
☐	☒	For null hypothesis testing, the test statistic (e.g. *F*, *t*, *r*) with confidence intervals, effect sizes, degrees of freedom and *P* value noted<br>*Give P values as exact values whenever suitable.*
☒	☐	For Bayesian analysis, information on the choice of priors and Markov chain Monte Carlo settings
☒	☐	For hierarchical and complex designs, identification of the appropriate level for tests and full reporting of outcomes
☐	☒	Estimates of effect sizes (e.g. Cohen's *d*, Pearson's *r*), indicating how they were calculated

Our web collection on statistics for biologists contains articles on many of the points above.

Software and code

Policy information about availability of computer code

Data collection	Data collection was conducted through google searches as described in the Methods section. All data sources (such as individual documents, online databases) are described in the main text and/or methods section and databases are also listed in the section "Data" below. There was no code used for data collection.
Data analysis	R Studio was used for the statistical analysis and for data visualisation. We used the following R version: R version 4.3.1 (2023-06-16) -- "Beagle Scouts". The custom code for calculation of avoided emissions has been deposited in a GitHub repository: https://github.com/poletresearch/coal_compensation.

For manuscripts utilizing custom algorithms or software that are central to the research but not yet described in published literature, software must be made available to editors and reviewers. We strongly encourage code deposition in a community repository (e.g. GitHub). See the Nature Portfolio guidelines for submitting code & software for further information.

Data

Policy information about availability of data

All manuscripts must include a data availability statement. This statement should provide the following information, where applicable:

- Accession codes, unique identifiers, or web links for publicly available datasets
- A description of any restrictions on data availability
- For clinical datasets or third party data, please ensure that the statement adheres to our policy

The compensation data generated in this study are available on Zenodo at 10.5281/zenodo.10782166. Coal power plant data has been retrieved from the S&P Global World Electric Power Plants Database. Data on IPCC pathways used in this study are available in the IPCC AR6 scenarios database (doi:10.5281/zenodo.5886912). Data on coal phase-out commitments used in this study are available in Vinichenko et al (2023) (DOI 10.1088/1748-9326/acadf6). Data on coal-based power generation used in this study are available in the IEA World Energy Balances database (doi:10.1787/data-00512-en). Data on amount of coal mined are available in the Enerdata database (https://yearbook.enerdata.net/coal-lignite/coal-production-data.html). Employment factors for coal power generation and coal mining used in this study are available in Pai et al (2021) (https://doi.org/10.1016/j.oneear.2021.06.005). Data on national GDP and inflation used in this study are available in the IMF World Economic Outlook database (we used the October 2022 version - https://www.imf.org/en/Publications/WEO/weo-database/2022/October), and data on GDP per capita adjusted for Purchasing Power Parity used in this study are available in the Penn World Table (DOI: 10.1257/aer.20130954). Data on government effectiveness used in this study is available in the World Bank's Worldwide governance indicators database (we used data from 2023 - https://www.worldbank.org/en/publication/worldwide-governance-indicators). Data on Hanson and Sigman's Index are available in Hanson and Sigman (2021) (https://doi.org/10.1086/715066). Data on Official Development Assistance is available from the OECD (we used data from 2023 - https://stats.oecd.org/Index.aspx?DataSetCode=TABLE2A). Data on coal production subsidies used in this study are from the OECD (https://www.oecd.org/fossil-fuels/countrydata/) and the IISD (https://odi.org/en/publications/g20-coal-subsidies-tracking-government-support-to-a-fading-industry/). Any other supporting data is publicly available and cited in the article. Source data for the figures and tables in the main text are provided with the article.

Research involving human participants, their data, or biological material

Policy information about studies with human participants or human data. See also policy information about sex, gender (identity/presentation), and sexual orientation and race, ethnicity and racism.

Reporting on sex and gender	N/A
Reporting on race, ethnicity, or other socially relevant groupings	N/A
Population characteristics	N/A
Recruitment	N/A
Ethics oversight	N/A

Note that full information on the approval of the study protocol must also be provided in the manuscript.

Field-specific reporting

Please select the one below that is the best fit for your research. If you are not sure, read the appropriate sections before making your selection.

☐ Life sciences ☒ Behavioural & social sciences ☐ Ecological, evolutionary & environmental sciences

For a reference copy of the document with all sections, see nature.com/documents/nr-reporting-summary-flat.pdf

Behavioural & social sciences study design

All studies must disclose on these points even when the disclosure is negative.

Study description	We conduct a mixed-methods study of countries which have time-bound coal phase-out pledges, further focusing on countries that have pledged compensation packages (publicly-financed financial transfers to support negatively affected actors including workers of coal power plants and mines; companies which are affected by coal phase-out; and countries and regions dependent on coal assets). We analyse the amounts, beneficiaries, and cost of compensation packages based on policy documents, news reports and other documents outlining the pledged policies. We conduct a regression analysis of the best predictors of compensation for coal phase-out and estimate the levels of compensation needed for coal phase-out in China and India in line with scenarios from the IPCC AR6.
Research sample	The population of relevant cases for our study includes all countries with time-bound coal phase-out pledges. We focus on this set of countries in order to better understand the relationship between financial compensation and coal phase-out ambition. We identify 43 such countries, 23 of which have compensation policies for coal phase-out. South Africa also receives compensation under its Just Energy Transition Partnership, but does not have a national coal phase-out pledge. Our quantitative analysis covers all cases in our population for which we could quantify compensation levels. Out of the 24 countries with compensation policies, we were able to

quantify the level of compensation in 21 countries (i.e. all except Northern Macedonia, Chile and Ukraine, where the Russo-Ukraine war made the implementation of coal phase-out policies very uncertain).

Sampling strategy	Our sample includes all countries in the relevant population for our study – those with time-bound coal phase-out commitments, based on Vinichenko et al (2023) (DOI 10.1088/1748-9326/acadf6).
Data collection	Data collection to identify all countries with coal phase-out compensation policies occurred through a systematic google search (Methods). Data were stored on computers (in clouds accessible to all researchers on the team, including Sharepoint). The researcher conducting the data collection was aware of the premise of the research.
Timing	Data for this study were first collected from August 2021 until November 2022. Data collection resumed from the receipt of the reviewers' comments in June 2023 until August 2023, and then again from December 2023 to January 2024.
Data exclusions	We excluded Ukraine from our analysis since even though it declared coal phase-out in 2020 and specified costs of compensation to coal companies in its 2022 budget, the start of the war in February 2022 made implementation of these plans highly uncertain. In our analysis of compensation policies, we excluded two line items for which the situation has substantially changed since the compensation policy was announced: (1) for Germany we excluded potential compensation to electricity consumers dependent on future electricity price changes due to the coal phase-out since this pledge was made prior to the Russo-Ukrainian war which made it difficult to quantify how much of the support for consumers could be attributed to coal phaseout and how much to policy measures in response to the energy security crisis; and (2) for the Netherlands we excluded requests for compensation from two coal power plant owners since they have been struck down by the courts. We exclude South Africa from our regression analysis and from our analysis of average compensation per ton of avoided CO2 emissions and coal capacity because the country does not have a coal phase-out date. We exclude Brunei Darussalam from our regression analysis because no data on governance are available.
Non-participation	Since there were no human participants, no participants dropped out or declined to answer.
Randomization	For our statistical analysis, we did not divide countries into groups but instead included all cases in our statistical analysis.

Reporting for specific materials, systems and methods

We require information from authors about some types of materials, experimental systems and methods used in many studies. Here, indicate whether each material, system or method listed is relevant to your study. If you are not sure if a list item applies to your research, read the appropriate section before selecting a response.

Materials & experimental systems

n/a	Involved in the study	
☒	☐	Antibodies
☒	☐	Eukaryotic cell lines
☒	☐	Palaeontology and archaeology
☒	☐	Animals and other organisms
☒	☐	Clinical data
☒	☐	Dual use research of concern
☒	☐	Plants

Methods

n/a	Involved in the study	
☒	☐	ChIP-seq
☒	☐	Flow cytometry
☒	☐	MRI-based neuroimaging

Plants

Seed stocks	N/A
Novel plant genotypes	N/A
Authentication	N/A
